# Evaluation of a Theory-Informed Implementation Intervention for the Management of Acute Low Back Pain in General Medical Practice: The IMPLEMENT Cluster Randomised Trial

**DOI:** 10.1371/journal.pone.0065471

**Published:** 2013-06-13

**Authors:** Simon D. French, Joanne E. McKenzie, Denise A. O'Connor, Jeremy M. Grimshaw, Duncan Mortimer, Jill J. Francis, Susan Michie, Neil Spike, Peter Schattner, Peter Kent, Rachelle Buchbinder, Matthew J. Page, Sally E. Green

**Affiliations:** 1 School of Public Health and Preventive Medicine, Monash University, Victoria, Australia; 2 Centre for Health, Exercise and Sports Medicine, The University of Melbourne, Melbourne, Victoria, Australia; 3 Clinical Epidemiology Program, Ottawa Hospital Research Institute, Ottawa, Canada; 4 Department of Medicine, University of Ottawa, Ottawa, Canada; 5 Centre for Health Economics, Monash University, Victoria, Australia; 6 Division of Health Services Research & Management, City University London, London, United Kingdom; 7 Centre for Outcomes Research and Effectiveness, University College London, London, United Kingdom; 8 Department of General Practice, The University of Melbourne, Melbourne, Victoria, Australia; 9 Victorian Metropolitan Alliance General Practice Training, Victoria, Australia; 10 Department of General Practice, School of Primary Health Care, Monash University, Victoria, Australia; 11 Research Department, Spine Centre of Southern Denmark, Middelfart, Denmark; 12 Institute of Regional Health Services Research, University of Southern Denmark, Odense, Denmark; 13 Monash Department of Clinical Epidemiology, Cabrini Health, Victoria, Australia; 14 Department of Epidemiology and Preventive Medicine, School of Public Health and Preventive Medicine, Monash University, Victoria, Australia; University of Michigan, United States of America

## Abstract

**Introduction:**

This cluster randomised trial evaluated an intervention to decrease x-ray referrals and increase giving advice to stay active for people with acute low back pain (LBP) in general practice.

**Methods:**

General practices were randomised to either access to a guideline for acute LBP (control) or facilitated interactive workshops (intervention). We measured behavioural predictors (e.g. knowledge, attitudes and intentions) and fear avoidance beliefs. We were unable to recruit sufficient patients to measure our original primary outcomes so we introduced other outcomes measured at the general practitioner (GP) level: behavioural simulation (clinical decision about vignettes) and rates of x-ray and CT-scan (medical administrative data). All those not involved in the delivery of the intervention were blinded to allocation.

**Results:**

47 practices (53 GPs) were randomised to the control and 45 practices (59 GPs) to the intervention. The number of GPs available for analysis at 12 months varied by outcome due to missing confounder information; a minimum of 38 GPs were available from the intervention group, and a minimum of 40 GPs from the control group. For the behavioural constructs, although effect estimates were small, the intervention group GPs had greater intention of practising consistent with the guideline for the clinical behaviour of x-ray referral. For behavioural simulation, intervention group GPs were more likely to adhere to guideline recommendations about x-ray (OR 1.76, 95%CI 1.01, 3.05) and more likely to give advice to stay active (OR 4.49, 95%CI 1.90 to 10.60). Imaging referral was not statistically significantly different between groups and the potential importance of effects was unclear; rate ratio 0.87 (95%CI 0.68, 1.10) for x-ray or CT-scan.

**Conclusions:**

The intervention led to small changes in GP intention to practice in a manner that is consistent with an evidence-based guideline, but it did not result in statistically significant changes in actual behaviour.

**Trial Registration:**

Australian New Zealand Clinical Trials Registry ACTRN012606000098538

## Introduction

Evidence-based clinical practice guidelines are a potential means of facilitating implementation of research into practice. However, what is not known is the best way to improve uptake of guidelines into practice. Previous studies have demonstrated that implementation interventions, that is, interventions designed to improve the uptake of evidence into practice, are effective some of the time. However we have little information to guide the choice of intervention to promote the uptake of guidelines for any given implementation problem [1]. Previous evaluations of implementation interventions have been criticised for not providing enough detail on the rationale for the developed intervention, including the hypothesised change processes that the intervention is targeting, or other relevant contextual factors [2].

Low back pain (LBP) is a common and costly condition. Estimates of the lifetime prevalence of LBP range from 6% to 66% (median 39%) [3]. At any one time, one in every four Australians have LBP, and four out of five Australians will experience it at some time in their lives [4]. Similar rates of LBP are reported in other high income countries around the world [5]. LBP carries a substantial financial burden, with the direct and indirect cost of LBP in Australia in 2001 estimated to be AUD$9,175 million [6].

Many people with LBP seek care from a general medical practitioner (GP). Two Australian national surveys of people with LBP have shown that of the 45% of people with acute LBP who sought care from a healthcare provider, they most commonly sought care from a GP (between 22% and 30% of those who sought care) [7], [8]. LBP is the second most common symptom, after a cough, for a visit to an Australian GP [9].

Management of LBP in general practice in Australia is not always concordant with recommended evidence-based practice [10], [11]. Plain film x-rays, and other imaging, for acute non-specific LBP are of limited diagnostic value, expose people to unnecessary ionising radiation, and provide no benefits in physical function, pain, or disability [12], [13]. However, plain film x-rays remain over utilised in the management of acute LBP in primary care settings [14], [15]. In Australia, over one quarter of patients with acute LBP receive an x-ray [10], [16] and over-utilisation of plain film x-rays has also been reported in the United States and Europe [13], [17]–[22]. Giving advice to stay active to people with acute LBP is supported by high level evidence and results in small benefits to patient outcomes [23], but it is underused by Australian GPs [10].

In 2003, the Australian National Health and Medical Research Council (NHMRC) endorsed an evidence-based guideline for the management of acute musculoskeletal pain [12]. The guideline was distributed by post to every Australian registered primary healthcare practitioner, and was available electronically on the NHMRC website. The aim of the guideline was to inform primary care practitioners of the evidence-based management of acute musculoskeletal pain, including non-specific LBP. We chose two key guideline recommendations (specifying recommended clinical behaviours, hereafter referred to as target behaviours) to address in this trial that were underpinned by high level evidence; one target behaviour related to diagnosis, that plain film x-rays were necessary only if fracture is suspected, and one related to treatment, that of providing advice to stay active, including the avoidance of advising more than two days of bed rest.

There have been a number of randomised trials evaluating interventions that aimed to improve the management of acute LBP in primary care settings [24]–[38]. These trials evaluated complex interventions of various combinations including educational outreach, educational workshops, and distribution of educational materials. The trials had varying success in changing certain practitioner behaviours and provide some information about methods of reducing x-ray referral for acute LBP in particular. However, these studies do not provide information about successful change of the behaviour of giving advice to stay active. For the implementation interventions evaluated in these studies, no explicit rationale was reported for the intervention chosen, and none reported the use of behavioural theory to design their intervention. More research is required to build on these studies in light of recent developments in implementation research. For example, current guidance recommends that interventions designed to increase the uptake of guidelines into clinical practice should address barriers and drivers of practice change [39]. Also, it is recommended that explicit theory is used to understand barriers, design interventions, and explore mediating pathways and moderators to advance the science of implementation research [40].

This study aimed to test the effectiveness and cost-effectiveness of a theory-informed intervention for implementing two behaviours recommended in a clinical practice guideline for acute LBP in general medical practice in Victoria, Australia. In this study we were unable to recruit sufficient patients to measure our original primary outcomes so we introduced other outcomes measured at the general practitioner (GP) level. A discussion of the difficulties we encountered in recruiting patient participants is published elsewhere [41]. This current paper reports an overview of the methods of this study, a description of the changes made to the planned outcome measures, and the effectiveness results. The full cost effectiveness results will be reported elsewhere.

## Materials and Methods

The trial protocol for IMPLEMENT (IMPLEmenting a clinical practice guideline for acute low back pain evidence-based manageMENT) is published elsewhere [42] and available as supporting information (Protocol S1). An overview of the methods is provided here, along with information about necessary deviations from the planned methods outlined in the protocol. The CONSORT checklist is available as supporting information (Checklist S1).

### Ethics Statement

Ethical approval for this trial was obtained from the Monash University Standing Committee on Ethics in Research involving Humans (2006/047). All participants provided written informed consent.

### Trial Design

This study was a cluster randomised trial, with the clusters being general medical practices including one or more GPs. The reasons for using this trial design were discussed in detail in the protocol [42].

### Recruitment of Practices and Patients

Recruitment of general practices occurred between October 2006 and May 2007. We approached all general medical practices in Victoria, Australia, via mail. GPs at these practices received a postcard with a short description of the study, followed by an invitation letter. A random selection of GPs was also contacted by telephone, with the number contacted in this way limited by available resources. To raise awareness of the study we placed notices about the study in relevant professional newsletters. When one GP in a practice agreed to participate and the practice was included, we then sent follow-up letters to the other GPs in the same practice informing them that the practice was included, encouraging them to participate, and allowing them to object to the practice participating if they wished. Included GPs were offered continuing professional development credits, or continuing medical education points, and access to LBP experts.

Patient participant recruitment commenced in November 2007 and ceased in June 2008. A full description of our attempted patient participant recruitment methods is published elsewhere [41]. Patient participant inclusion criteria were people presenting with acute (less than three months duration) non-specific LBP and aged 18 years or older. Exclusion criteria were described in the protocol [42]. We were unable to recruit a sufficient number of patient participants to measure the patient outcomes described in the protocol [42].

### Randomisation and Allocation Concealment

General practices were randomly allocated to either the intervention group or control group. A statistician independent of the study implemented the randomisation at a single point in time, and was only provided with general practice identification codes and stratification variables. Four stratification variables were defined by the number of GPs per practice (two levels: one to three GPs; four or more GPs) and whether the practice was in a rural or metropolitan location, defined as per Australian government geographical classification [43]. Within stratum, practices were allocated to the intervention and control groups with equal probability (1∶1 randomisation ratio) using computer-generated random numbers. Allocation was concealed from the investigators until baseline data had been collected from GPs.

### Blinding

Investigators (not involved in the delivery of the intervention), researcher assistants who entered the data and the statistician were blinded to group allocation until the statistical analysis was completed. Due to the nature of the intervention, it was not possible to blind the GPs to group allocation.

### Interventions

The control group received access to the guideline as per the guideline’s existing dissemination strategy, a printed copy of the guideline and a written reminder of how to access the electronic version of the guideline. Control group materials were sent to GPs in August 2007.

The process for developing the theory-informed intervention and a detailed description of the content of the intervention are published elsewhere [2]. Briefly, to develop the IMPLEMENT intervention, we used the Theoretical Domains Framework [44]–[46] of behaviour change to identify the barriers and enablers to the target behaviours and guide the choice of intervention components. Barriers to, and enablers of, the two target behaviours were identified in a qualitative study consisting of focus group interviews with 42 GPs in Victoria, Australia [47]. We then selected behaviour change techniques, to overcome these barriers and enhance the enablers, using a mapping tool designed for this purpose [39]. The selected behaviour change techniques included information provision, persuasive communication, provide information on consequences, provide opportunities for social comparison, barrier identification, model/demonstrate the behaviour, role play, provide instruction, time management and action planning. These techniques were combined to form a cohesive intervention delivered via two facilitated, interactive, educational workshops, each of three hours’ duration.

Workshops were a combination of didactic lectures and small group discussions and activities. We also produced a DVD to distribute to all GPs in the intervention group with the primary purpose of providing the material to those who could not attend the workshops. This alternative mode of delivering the same intervention content and messages included film footage from the workshops and electronic resources related to acute LBP management. The workshops were held between June and September 2007. The workshop content was evaluated by the Royal Australian College of General Practitioners, and the Australian College of Rural and Remote Medicine, and both organisations allocated continuing professional development points for GPs who attended both the workshops.

### Outcomes

The outcomes we measured in the trial deviated from those reported in the protocol because we were unable to recruit adequate numbers of patients to measure patient outcomes. Subsequent changes from the protocol for outcomes collected in the trial are outlined in Table 1.

**Table 1:** Planned outcomes from protocol [42] and outcomes actually measured.

Outcome	Planned in protocol	Collected in trial	Data collection method	When collected
**GP level**				
X-ray referral; Any imaging referral^1^	Y	N	Data abstraction from patient files	N/A
Advice to stay active; Advised bed rest^1^	Y	N	Telephone interview of patient participants	N/A
Behavioural constructs (e.g. knowledge, attitudes and intentions): Manage without x-ray referral;Give advice to stay active	Y	Y	Questionnaire	Baseline, 12 months
Fear Avoidance Beliefs (FAB)	Y	Y	Questionnaire	Baseline, 12 months
Behavioural simulation: X-ray referral; Anyimaging referral; Advice to stay active; Advice regardingbed rest	N	Y	Patient vignettes (Questionnaire)	12 months
X-ray and CT rates per patient seen	N	Y	Administrative data (Medicare imaging data)	12 months
**Patient level** 1				
Pain and Disability, FAB, Quality of Life andHealth Service Utilisation Items	Y	N	Telephone interview	N/A
X-ray occurred	Y	N	Telephone interview	N/A

1 Patient outcomes, and GP level outcomes measured at the patient level, were not collected because insufficient patient participants were recruited to the trial.

A questionnaire was distributed to GPs 12 months after intervention/control delivery (August 2008) that measured behavioural constructs, behavioural simulation (clinical decisions in response to simulated patient encounters as described in vignettes), fear avoidance beliefs and, for intervention GPs, whether they watched the intervention DVD. This questionnaire and scoring key is available as supporting information (see Questionnaire S1). Double data entry was undertaken for the GP questionnaires by research assistants and any discrepancies were resolved by consensus or by consultation with one of the investigators.

#### Behavioural constructs

Behavioural constructs (knowledge, attitude, beliefs and intention) considered to be predictors of behaviour [48], [49] were measured by a questionnaire we developed for the two target behaviours, managing patients without referral for x-ray and advising patients to stay active. Specific item content was informed by focus group interviews with 42 GPs conducted as part of this study [47]. The interviews used standard elicitation methods and covered GP views and experiences about the two target behaviours. Responses were used to design items measuring the theoretical constructs considered relevant to behaviour (drawn from the Theoretical Domains Framework and the Theory of Planned Behaviour) [44], [50], [51].The constructs included: behavioural intention (whether the GP intends to engage in the behaviour), attitude (whether the GP is in favour of performing the behaviour), subjective norm (how much the GP feels social pressure to engage in the behaviour), perceived behavioural control (whether the GP feels in control of the behaviour), beliefs about capabilities (whether the GP is confident in performing the behaviour), beliefs about professional role (whether the GP feels it is their professional responsibility to perform the behaviour), knowledge (whether the GP has knowledge about the behaviour), memory (whether the GP remembers to perform the behaviour) and environmental context (whether the GP feels the environmental context supports performance of the behaviour).

The questionnaire we developed to measure behavioural constructs contained 51 items and produced summary scores for the various behavioural constructs for each target clinical behaviour (see Questionnaire S1). There were 24 items related to providing advice to stay active; 22 of these were scored using a 7-point Likert scale, one item required a yes/no response and one item was multiple choice (later collapsed to a dichotomous scale).

There were 27 items relating to x-ray referral; 25 of these were scored using a 7-point Likert scale, one item recorded a yes/no response and one item was multiple choice collapsed to a dichotomous scale (adherent with guideline recommendation or not). Higher scores reflected greater intention to perform the target behaviour, more favourable attitudes toward the behaviour, greater social pressure to perform the behaviour, a greater level of perceived control over the behaviour, stronger belief in own capabilities of performing the behaviour, stronger belief that performance of behaviour is part of one’s professional role, greater knowledge about the behaviour, better memory for performing behaviour, and stronger belief that the environmental context supports performance of the behaviour. Different constructs were measured using between one and six items.

The psychometric properties (construct validity, test-retest reliability and predictive validity) of the behavioural constructs questionnaire were investigated in a separate sample of 528 Australian GPs prior to the trial (Unpublished data: O’Connor D, Monash University, Australia). Confirmatory factor analysis was conducted using LISREL v 8.7 to examine the construct validity of the tool. Test-retest reliability was investigated by calculating intra-class coefficients for the behavioural constructs for 98 GPs completing the survey on two occasions, separated by a two-week interval. Predictive validity was examined by interviewing a random subset of 100 GPs by phone one month after survey completion to record their behaviour and multiple regression analyses examined whether the behavioural constructs predicted behaviour. Factor analysis confirmed the hypothesised factor structure with all but one item demonstrating high factor loadings (>0.6) to the constructs which they intended to measure. Fit indices were high indicating good fit of the data to the model. Intra-class correlation coefficient values for the behavioural constructs ranged from 0.56 to 0.72 indicating moderate-to-good test-retest reliability. Multiple regression analyses demonstrated that the constructs *Attitudes*, *Subjective norms* and *Perceived behavioural control* were predictive of intention for both behaviours. The model explained 68% of the variance for intention to manage without referral for x-ray when the direct measures were used in the model (R2 adj = 0.68, F(3, 518) = 366.18, p<0.001) and explained 74% of the variance when indirect measures were added to the model in step 2 (R2 adj = 0.74, F change = 37.63, p<0.001). The model explained 52% of the variance for intention to advise patients to stay active when the direct measures were used (R2 adj = 0.52, F(3, 518) = 190.03, p<0.001) and explained 60% of the variance when indirect measures were added in step 2 (R2 adj = 0.60, F change = 33.09, p<0.001). Intention was predictive of the behaviour managing patients without an x-ray and univariate analysis showed that for every 1 unit increase in intention, the odds of managing patients without x-ray would increase by 40% of what they were. We were unable to examine the predictive validity of the intention-behaviour relationship for advice to stay active because all GPs interviewed after one month of completing the survey said they gave this advice to their patients.

GPs’ fear avoidance beliefs about LBP were measured via a modified version of the Fear-Avoidance Beliefs Questionnaire (FAB-Q) physical activity subscale [52] adapted for the GP participants [53]. The original questionnaire was designed for use with people with LBP, and we modified the items so that it can apply to practitioners, for example “Physical activity makes my pain worse” was modified to “Physical activity makes acute non-specific low-back pain worse”. The reliability and validity of these modifications was not evaluated. GPs’ fear-avoidance beliefs were included as an outcome because there have been some studies suggesting that practitioners’ fear-avoidance beliefs influence their treatment recommendations such that they are less likely to adhere to the guideline recommendations regarding giving advice to stay active [54]. The instrument consisted of four items, each measured on a 7-point Likert scale (0 = completely disagree, 6 = completely agree). Scores from these items were summed providing a score ranging from 0 to 24, with higher values representing greater fear-avoidance beliefs.

#### Behavioural simulation outcomes (clinical decision in response to vignettes)

Our behavioural simulation measures were also included in the questionnaire distributed to GPs 12 months post intervention/control delivery (see Questionnaire S1). These measures used four patient vignettes to simulate clinical decision-making about acute LBP management in specific situations. Elements of each scenario were drawn from the guideline [12], from focus groups conducted as part of this study [47], from another study in Victoria, Australia that evaluated a media campaign for LBP [55] and from the United Kingdom NEXUS study [56]. The patient vignettes were piloted with five practising GPs who were not part of the study.

For the behavioural simulation outcomes, adherence was determined regarding imaging and advice to stay active. X-ray adherence was defined as the GP not referring for a lumbosacral plain x-ray. Imaging adherence was defined as the GP not referring for any of following three diagnostic tests: lumbosacral plain x-ray, lumbar CT scan and lumbar magnetic resonance imaging (MRI). Activity adherence was defined as “Advise the patient to continue with their normal daily activities” regardless of other interventions selected (see Questionnaire S1 for other options available). Bed rest adherence was defined as either not recommending bed rest, or recommending bed rest for 2 days or less.

#### Referral rates for plain x-ray and CT scan (Administrative data outcomes)

GP plain film x-ray and CT scan referral were measured via medical administrative data (Australian Medicare data). Medicare is Australia's publicly funded universal healthcare system that provides free or subsidised care by health professionals such as GPs, including x-rays and other imaging. The large majority (approximately 95%) of patients who consult an Australian GP would have their imaging costs covered by Medicare [9]. The exceptions are injured workers and people who have sustained an injury as a result of an automobile accident, who are funded under different compensation systems. For each consenting GP, we obtained the total number of Medicare-funded patients who consulted the GP, and the number of low back-related imaging (x-ray and CT) referrals made by the GP in the period 12 months post intervention delivery. It was not possible to obtain data that was specifically linked to patients that presented with acute non-specific LBP. These data were likely to have included GP patients with chronic LBP and those where an x-ray may have been appropriate, e.g. a fracture was suspected.

### Sample Size

The original sample size calculations were based on measuring process (x-ray referral) and health (LBP-specific disability) outcomes on a cohort of patient participants recruited through the participating general practices. We determined that recruiting an average of 25 patient participants from each of the 92 general practices, providing a total of 2,300 patient participants, would be sufficient to detect important differences between groups in these outcomes with at least 90% power. Important differences were determined to be an absolute risk reduction of 10% in the percentage of patients referred for x-ray, assuming an x-ray referral rate of 20% in the control group, and a difference of at least two points in the Roland-Morris Disability Questionnaire, assuming a standard deviation of 6.0 (full details available in protocol [42], see Protocol S1). However, we only recruited 29 patient participants in total during the recruitment period.

### Analyses

#### Statistical analysis

The effects of the intervention were estimated with marginal models using Generalised Estimating Equations (GEEs), with robust variance estimation (sandwich variance estimator), to account for the correlation of responses within practices. An exchangeable correlation structure was specified, where responses from the same practice were assumed to be equally correlated. However, when the estimated intra-cluster correlation coefficient (ICC) from the model was negative, the model was refitted with an independent correlation structure, that is, assuming an ICC of zero. This approach yields conservative estimates of standard errors and follows the recommendations and practice of others in assuming that in this context, the likely explanation for a negative ICC is sampling variability, not a true underlying negative ICC (e.g. [57]–[59]). However, refitting the models with an independent correlation structure made a negligible difference to the estimated standard errors.

Models were adjusted for design strata and confounders, which were specified prior to undertaking the analysis. The confounders included in each model are noted in the footers of the results tables. Results from these models were compared with those which only adjusted for design strata (results available in Tables S1, S2 and S3 in File S1). Marginal logistic and negative binomial regression models were used for binary and count outcomes respectively. The negative binomial heterogeneity parameter used in the marginal model was first estimated from fitting a generalised linear negative binomial model.

The measure of intervention effect arising from the marginal logistic models is an odds ratio. To aid interpretation, we planned to transform the odds ratios to risk ratios using the method of Zhang et al 1998 [60]. However, updated CONSORT guidance recommends that both a measure of absolute and relative effect should be provided [61]. We therefore used a more general approach of calculating risk ratios and risk differences from marginal probabilities estimated from the fitted logistic models [62]. Confidence intervals for the effect measures were estimated using bootstrap methods, allowing for clustering of observations within general practices. Bias corrected 95% confidence intervals were calculated from 1000 replicates. Sensitivity analyses were undertaken to compare how the effect measures computed from the logistic models compared with other methods proposed in Zhang et al 1998 [60], [63], and McNutt et al 2003 [64]. All methods yielded similar estimates and confidence limits.

Imaging (x-ray and CT-scan) referral was analysed as a binary variable for the purpose of calculating ICCs (as opposed to a count variable aggregated at the level of the GP). Point estimates of the ICCs were estimated from analysis of variance. Confidence intervals for the ICCs were bootstrapped using the combination of the bootstrap and loneway commands in Stata version 12 [65]. Bootstrapping allowed for clustering of observations within general practices. Bias corrected 95% confidence intervals were calculated from 1000 replicates.

We had planned to undertake a per-protocol analysis to investigate the effect of the intervention for the subgroup of GP participants who actually attended the workshop [42]. However, given we were unable to collect data on the primary outcome of the trial, we decided not to undertake this analysis. No adjustment for multiple testing was undertaken. All models were fitted using the statistical package Stata version 12 [65].

## Results

All 1688 general practices in the state of Victoria, Australia, were invited to participate in the trial. GPs from 92 general practices agreed to participate and were randomised (45 practices to the intervention and 47 to the control group), including a total of 112 GPs. Practice and GP baseline characteristics are shown in Table 2. There was some baseline imbalance in GP characteristics with control group GPs more likely to identify themselves as having a special interest in LBP (24% versus 9%), and more GPs in the intervention group undertaking LBP continuing education in the past year (16% versus 5%). Baseline behavioural constructs and fear-avoidance beliefs were similar between the groups (Table 3).

**Table 2:** General practice and general practitioner (GP) baseline characteristics.

	Intervention group		Control group	
**Practice factors at baseline**	*N practices*		*N practices*	
Number of practices	45		47	
Number of GPs per practice (SD)	45	5 (3.9)	47	5 (3.8)
Number of practices with 1, 2, and 3 participating GPs:	45		47	
- 1 GP participated		35		41
- 2 GPs participated		6		6
- 3 GPs participated		4		0
No. (%) rural practices	45	15 (33)	47	16 (34)
No. (%) with x-ray facility on site	43	3 (7)	46	1 (2)
No. (%) of industrial practices	44	3 (7)	46	4 (9)
No. (%) of training practices	43	30 (70)	46	27 (59)
Method of billing*:	41		44	
- No. (%) Bulk bill		7 (17)		6 (14)
- No. (%) Co-payment		34 (83)		38 (86)
**GP factors at baseline**	*N GPs*		*N GPs*	
Number	59		53	
Mean age (years) (SD)	59	50 (9.5)	52	53 (11.5)
No. (%) female	59	20 (34)	52	19 (37)
Mean number of years since graduated (SD)	59	26 (9.8)	52	29 (11.3)
No. (%) with special interest in LBP	56	5 (9)	45	11 (24)
No. (%) undertaken LBP continuing education in past year	56	9 (16)	44	2 (5)
Mean number of patients seen per week (SD)	56	123 (58.2)	44	130 (52.6)
Mean number of LBP patients seen per week (SD) (averaged over the previous month) [Median; IQR]	56	3 (4.0) [2; 1 to 3]	42	3 (4.7) [2; 1 to 3]
No. (%) who are members of local GP Division#	55	53 (96)	45	40 (89)

SD: standard deviation; No.: number; IQR: Interquartile range [25^th^ percentile to 75^th^ percentile]; LBP: low back pain.

* Bulk bill: the total payment for patient’s consultation is paid for by the Medicare system; Co-payment: Medicare system pays for part of the consultation and the patient pays for the remainder of the cost.

# The Divisions of General Practice Program was funded by the Australian Government to provide services and support to general practice.

**Table 3:** Baseline summary statistics of the behavioural constructs for the clinical behaviours ‘managing patients without referral for plain x-ray’ and ‘advising patients to stay active’^1^.

		Intervention group GPs (N = 56)^2^		Control group GPs (N = 45)	
Variable	Possible rangeof responses	Mean (SD)	Median (IQR)	Mean (SD)	Median (IQR)
**Managing patients without referral for plain x-ray**					
Behavioural intention (generalised)	1 to 7	5.4 (1.41)	5.7 (5.0 to 6.5)	5.5 (1.28)	6.0 (5.0 to 6.7)
Behavioural intention (performance)^3^	0 to 10	7.6 (2.79)	8.0 (7.0 to 10.0)	8.1 (1.98)	9.0 (6.0 to 10.0)
Attitude (direct)^4^	1 to 7	5.7 (1.17)	6.0 (5.1 to 6.8)	5.5 (1.19)	5.6 (4.6 to 6.4)
Attitude (indirect)^4^	1 to 7	4.9 (0.99)	4.8 (4.3 to 5.7)	4.7 (1.04)	4.7 (4.2 to 5.2)
Subjective norm (direct)	1 to 7	4.5 (1.14)	4.7 (4.0 to 5.0)	4.4 (1.38)	4.3 (3.3 to 5.0)
Subjective norm (indirect)	1 to 7	4.4 (1.05)	4.6 (3.6 to 5.2)	4.2 (1.14)	4.4 (3.6 to 4.8)
Perceived behavioural control (direct)	1 to 7	5.2 (1.29)	5.5 (4.1 to 6.3)	5.1 (1.30)	5.3 (4.5 to 6.0)
Perceived behavioural control (indirect)^5^	−42 to 42	−2.3 (10.45)	0.0 (−8.0 to 3.0)	−3.4 (8.65)	−3.0 (−9.0 to 0.0)
Beliefs about professional role	1 to 7	5.5 (1.20)	5.7 (4.7 to 6.3)	5.3 (1.20)	5.7 (4.7 to 6.0)
Knowledge (No. (%))	0 or 1	31 (55%)	–	26 (58%)	–
Beliefs about capabilities (red/yellow flags)	1 to 7	5.6 (1.04)	6.0 (5.0 to 6.0)	5.8 (1.01)	6.0 (5.0 to 7.0)
Beliefs about capabilities (reassure)	1 to 7	5.4 (1.20)	6.0 (5.0 to 6.0)	5.2 (1.40)	6.0 (5.0 to 6.0)
**Advising patients to stay active**					
Behavioural intention (generalised)	1 to 7	6.0 (0.95)	6.2 (5.7 to 7.0)	6.0 (1.16)	6.3 (5.3 to 7.0)
Behavioural intention (performance)^3^	0 to 10	8.9 (2.35)	10.0 (8.0 to 10.0)	8.8 (2.29)	10.0 (9.0 to 10.0)
Attitude (direct)	1 to 7	5.9 (1.28)	6.0 (5.6 to 7.0)	5.8 (1.40)	6.0 (5.4 to 7.0)
Attitude (indirect)	1 to 7	5.0 (0.73)	5.0 (4.5 to 5.5)	4.8 (0.82)	4.8 (4.3 to 5.2)
Subjective norm (direct)	1 to 7	4.6 (1.15)	4.7 (4.0 to 5.3)	4.7 (1.06)	5.0 (4.0 to 5.3)
Subjective norm (indirect)	1 to 7	4.5 (0.93)	4.5 (4.0 to 5.0)	4.7 (0.83)	4.5 (4.0 to 5.0)
Perceived behavioural control (direct)	1 to 7	5.5 (1.10)	5.8 (4.8 to 6.3)	5.6 (1.14)	5.8 (5.0 to 6.5)
Perceived behavioural control (indirect)	−63 to 63	14.3 (15.26)	12.5 (3.0 to 25.5)	14.3 (15.49)	15.0 (0.0 to 26.0)
Beliefs about professional role	1 to 7	6.2 (0.85)	6.5 (6.0 to 7.0)	6.3 (0.79)	6.5 (6.0 to 7.0)
Knowledge (No. (%))	0 or 1	37 (66%)	–	34 (76%)	–
Environmental context	1 to 7	5.8 (1.30)	6.0 (5.0 to 7.0)	5.7 (1.47)	6.0 (5.0 to 7.0)
Memory	1 to 7	4.8 (1.55)	5.0 (3.0 to 6.0)	5.0 (1.78)	5.0 (4.0 to 7.0)
Fear-avoidance beliefs	0 to 24	9.5 (3.95)	9.0 (7.0 to 12.0)	9.1 (4.58)	9.0 (6.0 to 12.0)

GPs: general practitioners; IQR: interquartile range; SD: standard deviation.

1 For all outcomes (except fear-avoidance beliefs) a larger score indicates greater agreement or likelihood in the practitioners’ intentions and beliefs in performing the particular behaviour (i.e. not referring for plain x-ray or advising patients to stay active). For example, a larger score on the behavioural construct “Attitudes (direct)” for managing patients without referral for plain x-ray indicates that the practitioner is more in favour of performing this behaviour. For the fear-avoidance beliefs scale, a larger score indicates greater fear avoidance beliefs surrounding physical activity and pain in acute non-specific low back pain.

2 Except for variables “perceived behavioural control (indirect)” and “fear-avoidance beliefs” where N = 55.

3 Behavioural intention performance for managing patients without referral for x-ray was reverse coded for consistency with the interpretation of the other behavioural constructs.

4 Construct measured directly (e.g. by asking GPs about their overall attitude) and indirectly (e.g. by asking about specific behavioural beliefs).

5 Perceived behavioural control (indirect) is a function of control belief items multiplied by control power items and can range from negative to positive values, with larger positive scores indicating greater perceived control over the behaviour.

**Figure 1:**
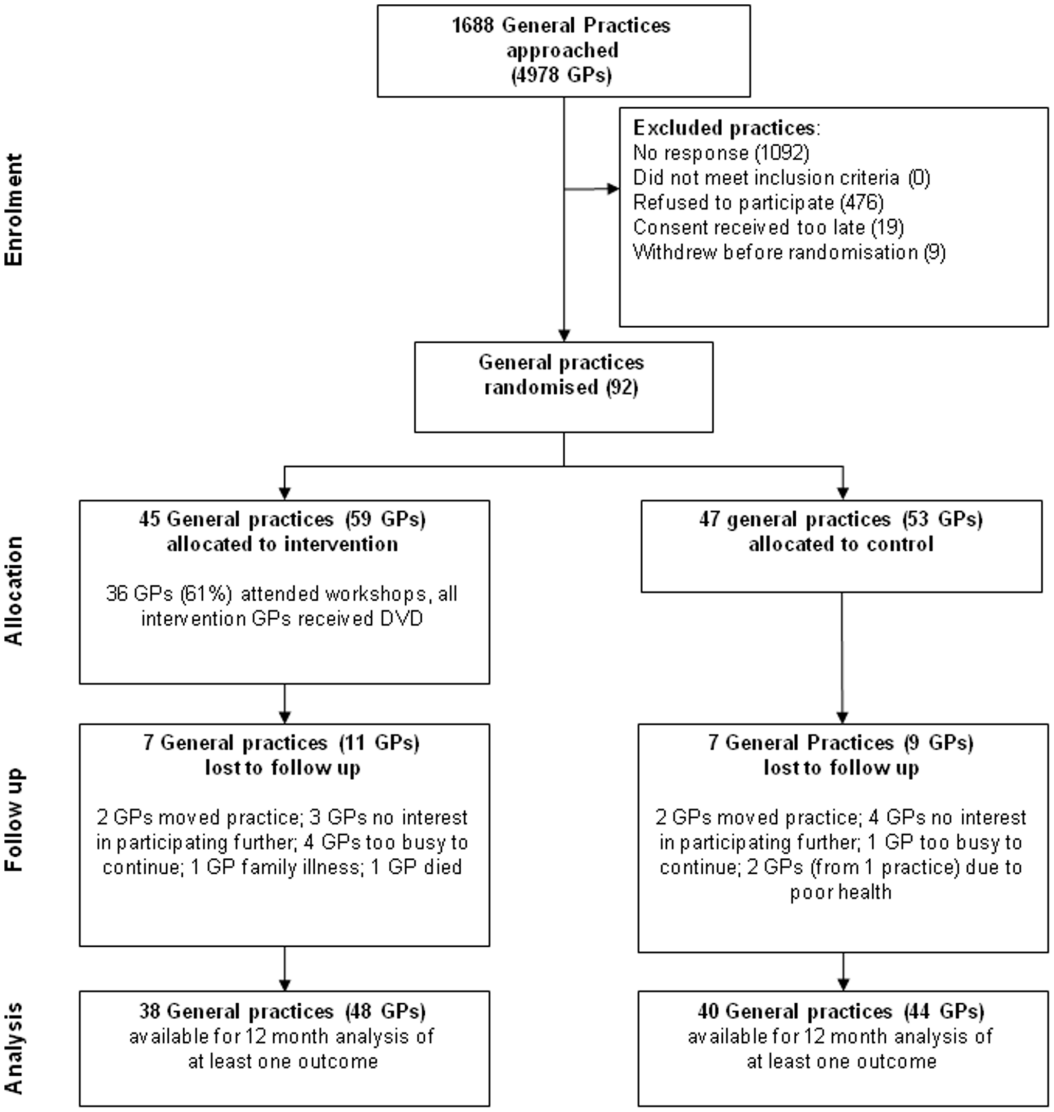
Flow of practices through the IMPLEMENT cluster randomised trial.

Flow of practices and GPs through the trial is shown in Figure 1. Overall 78 practices (85%) and 92 GPs (82%) were available at final follow up. However, the number of GPs available for analysis was less than 82% (ranging from 70% to 75%) due to missing confounder information (see results Tables for sample sizes). Thirty-six (61%) of the 59 GPs randomised to the intervention group attended the two facilitated workshops and all intervention GPs were sent the DVD. The main reason cited by the GPs who did not attend the workshops was lack of time. Fourteen GPs reported that they had watched the DVD (eight who attended and six who did not attend the workshops).

### Effectiveness of the Intervention at 12 Months Post Intervention

#### Behavioural construct outcomes

The intervention effect estimates for the behavioural construct outcomes are reported by theoretical domain (Table 4). For the clinical behaviour managing patients without an x-ray, there was a statistically significant difference in the behavioural constructs *Behavioural intention* (performance), *Attitude* (indirect) and *Beliefs about capabilities* (reassure). However, the intervention effects, while favouring the intervention, were typically small, and even the upper limit of the confidence intervals for outcomes measured on the 7-point Likert scale were less than 1 unit difference between groups. An exception to this was the measure of *Behavioural intention* (performance) where, on average, GPs in the intervention group indicated that out of their next 10 patients they would refer 0.8 (95%CI 0.4, 1.2) of a patient for x-rays less than the control GPs. For the clinical behaviour advising patients to stay active, there were no statistically significant differences between the groups for the behavioural construct outcomes. For intention measures for both clinical behaviours (generalised intention and performance intention), GPs in both groups reported high intention to manage patients in a manner consistent with the guidelines. GPs in the intervention group demonstrated lower fear-avoidance beliefs about physical activity at 12 months (adjusted difference in means −2.4, 95%CI −3.9 to -0.8; scale ranging from 0 to 24).

**Table 4:** Effect of the intervention on the behavioural constructs for the clinical behaviours 'managing patients without referral for plain x-ray' and 'advising patients to stay active'.

	Intervention group^1^	Control group^2^	Adjusted effect estimate^4^	(95% CI)	p-value
Variable	Mean (SD)^3^	Mean (SD)^3^			
**Managing patients without referral for plain x-ray**					
Behavioural intention (generalised)	6.2 (0.67)	5.8 (1.22)	0.3	(−0.1, 0.6)	0.180
Behavioural intention (performance)	9.4 (0.77)	8.6 (1.30)	0.8^*^	(0.4, 1.2)	0.000
Attitudes (direct)	6.2 (1.00)	5.8 (0.99)	0.4	(−0.0, 0.9)	0.055
Attitudes (indirect)	5.4 (0.84)	4.8 (0.97)	0.4^*^	(0.1, 0.7)	0.018
Subjective norms (direct)	4.7 (0.88)	4.6 (1.38)	−0.2^*^	(−0.5, 0.2)	0.425
Subjective norms (indirect)	4.9 (0.86)	4.5 (1.04)	0.1^*^	(−0.2, 0.5)	0.457
Perceived behavioural control (direct)	5.3 (1.08)	5.3 (0.94)	0.1^*^	(−0.3, 0.4)	0.658
Perceived behavioural control (indirect)	1.4 (7.71)	−0.2 (7.91)	0.9^*^	(−2.2, 4.0)	0.558
Beliefs about professional role	5.9 (0.95)	5.4 (1.14)	0.3	(−0.1, 0.6)	0.176
Knowledge (No. (%))	31 (76%)	30 (71%)	1.15^5*^	(0.38, 3.46)	0.806
Beliefs about capabilities (red/yellow flags)	6.0 (0.95)	5.8 (1.23)	0.1^*^	(−0.3, 0.5)	0.643
Beliefs about capabilities (reassure)	6.0 (0.65)	5.6 (1.06)	0.3^*^	(0.0, 0.7)	0.039
**Advising patients to stay active**					
Behavioural intention (generalised)	6.4 (0.66)	6.0 (1.24)	0.1	(−0.2, 0.4)	0.410
Behavioural intention (performance)	9.5 (1.61)	9.0 (2.08)	0.4^*^	(−0.2, 0.9)	0.220
Attitudes (direct)	6.3 (1.04)	6.3 (0.98)	−0.1	(−0.4, 0.3)	0.801
Attitudes (indirect)	5.2 (0.73)	4.9 (0.83)	0.2^*^	(−0.1, 0.5)	0.177
Subjective norms (direct)	4.9 (0.98)	4.6 (1.15)	0.2^*^	(−0.1, 0.5)	0.241
Subjective norms (indirect)	4.7 (0.89)	4.4 (1.03)	0.3^*^	(−0.1, 0.6)	0.135
Perceived behavioural control (direct)	5.8 (0.81)	5.7 (0.97)	0.1^*^	(−0.2, 0.4)	0.373
Perceived behavioural control (indirect)	15.9 (16.66)	13.1 (13.93)	3.2	(−2.4, 8.8)	0.266
Beliefs about professional role	6.4 (0.62)	6.3 (0.73)	0.0	(−0.3, 0.3)	0.928
Knowledge (No. (%))	39 (95%)	38 (90%)	1.26^5^	(0.15, 10.59)	0.833
Environment context	5.7 (1.25)	5.7 (1.38)	0.1^*^	(−0.4, 0.5)	0.807
Memory	5.3 (1.50)	5.0 (1.62)	0.3	(−0.3, 0.8)	0.296
Fear-avoidance beliefs	7.6 (4.10)	10.0 (4.23)	−2.4^*^	(−3.9, −0.8)	0.004

1 No. Practices = 34 and No. GPs = 41, except for variable “Perceived behavioural control (indirect)” where No. Practices = 34 and No. GPs = 40.

2 No. Practices = 38 and No. GPs = 42, except for variable “Behavioural intention (performance)” where No. Practices = 37 and No. GPs = 41.

3 Only general practitioners who provided both baseline and follow-up measures of the behavioural constructs are included in the calculation of the means.

4 Adjusted difference in means estimated from marginal linear regression models using GEEs with an exchangeable correlation structure and robust variance estimation to allow for clustering within general practices. For models where the estimated within cluster correlation was negative (indicated by^*^), the model was refitted assuming an independent correlation structure. All models adjusted for the design strata (number of GPs per practice, location of practice [metropolitan or rural/remote]) and confounders specified prior to undertaking the analysis (age of GP (years), special interest in LBP, baseline measure of the behavioural construct).

5 Adjusted odds ratio estimated from marginal logistic regression models using GEEs with an exchangeable correlation structure and robust variance estimation to allow for clustering within general practices. For models where the estimated within cluster correlation was negative (indicated by^*^), the model was refitted assuming an independent correlation structure. Models adjusted for the same variables as in footnote 4.

#### Behavioural simulation outcomes (in response to vignettes)

At 12 months post-intervention, compared with GPs in the control group, GPs in the intervention group had larger odds of adhering to the guideline recommendations about x-ray and imaging, and advice regarding activity as measured in response to vignettes (Table 5). Table 6 provides odds ratios transformed to risk ratios and risk differences to aid interpretation. At follow-up GPs in both the intervention and control group almost always reported adherence to the guideline recommendations regarding advice to avoid bed rest (99% and 98% respectively).

**Table 5:** Effect of the intervention on adherence to the guideline for the clinical behaviours x-ray referral, imaging referral, advice regarding activity and bed rest, as measured by response to the vignettes (behavioural simulation outcomes).

Variable	Intervention group adherence^1^		Control group adherence^2^		Adjusted Odds Ratio^3^	(95% CI)	p-value
	No.	(%)	No.	(%)			
X-ray adherence^4^	126/152	(83)	109/160	(68)	1.76^*^	(1.01, 3.05)	0.045
Imaging adherence^4^	119/152	(78)	89/160	(56)	2.36^*^	(1.48, 3.79)	0.000
Activity adherence^5^	121/152	(80)	82/160	(51)	4.49	(1.90, 10.60)	0.001
Bed rest adherence^6^	163/164	(99)	168/171	(98)	2.91^*^	(0.30, 27.83)	0.354

1 No. Practices = 31 and No. GPs = 38, except for variable “Bed rest adherence” where No. Practices = 34 and No. GPs = 41.

2 No. Practices = 36 and No. GPs = 40, except for variable “Bed rest adherence” where No. Practices = 38 and No. GPs = 43.

3 Adjusted odds ratios estimated from logistic models fitted using GEEs with an exchangeable correlation structure and robust variance estimation to allow for clustering within general practices. For models where the estimated within cluster correlation was negative (indicated by ^*^), the model was refitted assuming an independent correlation structure.

4 Model adjusted for the design strata (number of GPs per practice, location of practice [metropolitan or rural/remote]) and confounders specified prior to undertaking the analysis (age of GP (years), years since GP graduated, special interest in LBP, practice method of billing [bulk bill or co-payment]).

5 Model adjusted for the same design strata and confounders as in footnote 4, with the addition of the baseline measure of GP fear-avoidance beliefs.

6 The planned model was to have included adjustment for the same design strata and confounders as in footnote 5, however; due to limited events of non-adherence the model was fitted with no adjustment for the design strata and confounders.

**Table 6:** Effect of the intervention on adherence to the guideline for the behaviours x-ray referral, imaging referral, advice re activity and bed rest, as measured by the vignettes, using different effect metrics.

Variable	Adjusted Risk Ratio^1,2^	(95% CI)	Adjusted Risk difference^1,2^	(95% CI)
X-ray adherence	1.14	(1.01, 1.29)	0.10	(0.00, 0.20)
Imaging adherence	1.30	(1.15, 1.50)	0.18	(0.09, 0.27)
Activity adherence	1.59	(1.21, 2.14)	0.30	(0.12, 0.44)
Bed rest adherence	1.01^3^	(1.00, 1.04)	0.01^3^	(−0.00, 0.04)

1 All models adjusted for design strata and confounders specified prior to undertaking the analysis. See Table 4 for details of the design strata and confounders. The exception to this was “Bed rest adherence” which, due to limited events of non-adherence, was fitted with no adjustment for design strata and confounders.

2 Metrics (RR and RD) calculated from marginal probabilities [62]. Confidence intervals for the metric were bootstrapped in Stata [65] allowing for clustering of observations within general practices (using both the *cluster()* and *idcluster()* options). Bias corrected 95% confidence intervals were calculated from 1000 replicates.

3 CI limits could only be calculated from 612 bootstrapped replicates.

#### Referral rates for plain x-ray and CT scan (administrative data outcomes)

Rates of x-ray referral in the intervention and control groups were 8.3 and 10.2 per 1000 patients respectively, and rates of CT-scan referral were 6.1 and 6.6 per 1000 patients respectively (Table 7). Incidence rate ratios of referral in the intervention group compared with the control were 0.83 (95%CI 0.61 to 1.12) for x-ray, 0.92 (95%CI 0.66, 1.27) for CT-scan, and 0.87 (95%CI 0.68, 1.10) for x-ray or CT-scan. All confidence intervals included an incidence rate ratio of one indicating no statistically significant difference in x-ray and CT referral between the groups. The potential importance of these effects was unclear.

**Table 7:** Effect of the intervention on imaging referral.

Variable	Intervention group follow-up^1^		Control group follow-up^2^		Incident rate ratios^3^	(95% CI)	p-value
	No. referrals	Rate/1000 patients	No. referrals	Rate/1000 patients			
X-ray referral	643	8.3	768	10.2	0.83*	(0.61, 1.12)	0.211
CT-scan referral	474	6.1	496	6.6	0.92	(0.66, 1.27)	0.598
X-ray or CT-scan referral	1117	14.4	1264	16.8	0.87	(0.68, 1.10)	0.244

1 No. Practices = 34 and No. GPs = 44; Total number of Medicare patients seen by GPs in intervention group = 77,716.

2 No. Practices = 37 and No. GPs = 40; Total number of Medicare patients seen by GPs in control group = 75,226.

3 Incident rate ratios estimated from negative binomial models fitted using GEEs with an exchangeable correlation structure and robust variance estimation to allow for clustering within general practices. For models where the estimated within cluster correlation was negative (indicated by^*^), the model was refitted assuming an independent correlation structure. All models adjusted for the design strata (number of GPs per practice, location of practice [metropolitan or rural/remote]) and confounders specified prior to undertaking the analysis (age of GP (years), years since GP graduated, special interest in LBP, practice method of billing [bulk bill or co-payment]).

#### Intra-cluster correlations (ICC)

Estimated ICCs for the administrative data outcomes were very small (0.004 (95%CI 0.003, 0.006) for x-ray referral and 0.003 (95%CI 0.002, 0.005) for CT-scan referral) indicating little evidence of clustering. Estimates of ICCs for behavioural simulation outcomes as measured via the vignettes were typically larger, except for advice regarding bed rest (which is likely to be explained by the very low prevalence of non-adherence) [59], [66]. A particularly large ICC was observed for advice to stay active (0.398 (95%CI 0.265, 0.550)), indicating variation in management of patients for this outcome across general practices. Since there were few practices with more than one GP, these results can also be interpreted as being reflective of management across GPs. See Table S4 in File S1 for full ICC results.

## Discussion

An intervention informed by evidence and a behaviour change theoretical framework, compared to simple guideline dissemination alone, had some influence on GP adherence to an evidence-based guideline for the management of LBP at 12 months post-intervention. For behavioural construct outcomes (*Behavioural intention, Attitude, Subjective norm, Perceived behavioural control, Beliefs about professional role* and *Beliefs about capabilities*) there was greater agreement or likelihood in the GPs’ intentions and beliefs in performing the clinical behaviour not referring for plain x-ray in line with the guideline recommendations, but the point estimates were typically small. For the clinical behaviour advising patients to stay active, there were no statistically significant differences between the groups for the behavioural construct outcomes. For behavioural simulation, the intervention improved GPs’ intended adherence to an evidence-based guideline as measured by patient vignettes at 12 months post-intervention. For lumbar imaging rates (administrative medical imaging data), there was no statistically significant difference in x-ray and CT referral rates between the groups. The potential importance of the intervention on lumbar imaging rates for those with acute non-specific LBP remains unclear since the administrative data was not specific to patients with this condition.

While these results suggest that the evaluated intervention holds some promise for improving guideline uptake, this intervention typically entails an upfront investment that may or may not be offset by any health gains and/or reductions in health service utilisation. The potential for improvements in clinical practice should therefore be weighed against the additional cost or cost-savings attributable to the intervention. Results from an economic evaluation alongside the IMPLEMENT trial, suggest that, after taking account of reductions in service use, the IMPLEMENT intervention may actually be less costly than standard dissemination. At the mean, the IMPLEMENT intervention dominated (less costly and more effective) standard dissemination for the outcomes of x-ray referral and intention to adhere to the guideline in behavioural simulation. Confidence intervals around point estimates of incremental cost-effectiveness for the outcome of x-ray referral suggest that we cannot be at least 95% confident that the IMPLEMENT intervention differs in value from standard dissemination. Confidence intervals around point estimates of cost-effectiveness for the outcome of intention to adhere to the guideline in behavioural simulation suggest that, given a sufficiently high willingness to pay, we can be at least 95% confident that the IMPLEMENT intervention represents good value in comparison to standard dissemination. Full results from the economic evaluation alongside the IMPLEMENT trial and a discussion of the limitations of the economic analysis will be reported elsewhere.

Estimated ICCs for the administrative imaging outcomes were very small. Several hypotheses may explain these results including the low prevalence of the outcomes [59], [66], a large amount of variability in the outcomes arising through the inclusion of all patients (not only patients with acute LBP), or consistency of management of patients across GPs. For the behavioural simulation outcome data, which sought GPs’ intended clinical decisions in relation to only acute LBP patients, ICCs of x-ray and imaging adherence were larger, indicating some inconsistency in the intended management of patients across GPs (although the confidence intervals did not exclude an ICC of zero). Of particular note was the large ICC for advice to stay active, indicating a lack of consistency in GP intended practice for this clinical behaviour. This uncertainty may arise from the GP community being unclear about what is meant by providing advice to stay active. Discussions with GPs in the intervention workshops indicated that interpretations of advice to stay active were variable, ranging from advising patients to go for a swim or a walk, to giving patients specific low back exercises, to advising patients to return to their normal activities as soon as possible. This issue may be related to the predominance of GPs taking a biomedical (or structural) orientation to LBP, which in some studies has been linked to advising patients to restrict activity [67]. GPs appear to need further education and resources about this aspect of care, and changing clinical behaviour relating to giving advice to stay active for patients with acute LBP should be considered in GP workforce education and policy.

There was no statistically significant difference in the behavioural simulation outcome between the groups for recommending bed rest. The vast majority of GPs from both groups adhered to this guideline recommendation and assuming the GPs in this study are representative of the primary care population, it appears few GPs would recommend bed rest to this acute LBP patient population.

For other aspects of evidence-based recommendations for acute LBP, it is evident that there are problems remaining in primary care with ongoing discordance with clinical practice guidelines [10], [11]. Questions remain about the best way to approach this. As in clinical research, testing a small number of interventions to address a problem does not guarantee a clear and immediate solution to the problem. We agree with the view now frequently argued in the literature [68], [69] that it is important to build a cumulative science of implementation (which relies in large part on a cumulative science of behaviour change) and we trust that the trial we report in this paper makes a contribution to that science.

### Comparison with Other Studies

This trial adds to the evidence-base in the implementation literature for LBP management in a number of ways. First, it shows that a theory-informed intervention, compared to simple guideline dissemination alone, can make small changes to GP intention to practise in a manner that is consistent with recommendations from an evidence-based guideline. Previous studies in this area have lacked strong rationale for intervention choice and have not assessed barriers and facilitators to uptake of evidence into practice prior to intervention development [70]. Like our study, previous implementation studies aimed at primary care providers to reduce x-ray utilisation and increase activity advice for acute LBP patients have demonstrated generally small effects, whether the outcomes were measured as process level outcomes or as patient outcomes [24]–[33], [38], [71], [72].

This trial included an economic evaluation that has been absent in most previous trials in this area. Unfortunately our inability to recruit sufficient patients into the trial has limited the full cost-effectiveness evaluation, however we were able to determine the costs of developing a theoretically-informed intervention and relate this to potential costs saved in the reduction of plain film x-ray utilisation.

Whether using theory as a basis for behaviour change intervention design will lead to more effective interventions is not well understood. Some reviews have concluded that the use of theory is associated with larger intervention effects [73]–[77], while other reviews have concluded small, none or negative association between reported theory use in intervention design and intervention effectiveness [78]–[80]. More research is required in this area, including studies to compare the use of different theories in developing interventions, or to compare the use of theory in developing interventions with other intervention development methods.

### Strengths and Weaknesses of the Study

The main strength of this study was that this is, to the authors’ knowledge, the first theoretically-informed implementation intervention to be tested in a cluster randomised trial design for the management of acute LBP in the general medical practice setting. The intervention was developed in light of recent thinking amongst implementation scientists that interventions designed to improve the uptake of evidence into practice require a strong rationale and need to be theoretically informed [2].

The major limitation of the study was that we were unable to recruit patient participants, and were therefore not able to measure actual GP clinical behaviours, patient health outcomes, patient behaviours, and patient level measures of health service utilisation. Nor could we measure practitioners’ management of patients in a cohort of acute LBP patients. Failure to recruit patient participants was in spite of multiple changes to our recruitment strategy [41]. We surveyed participating GPs about patient recruitment, and despite intending to recruit patients, GPs forgot to approach patients to participate and GPs were ambivalent about whether patients were interested, or not, in participating. In a busy general practice clinic with GPs consulting only a few acute LBP patients every week their ability to approach patients for recruitment was clearly difficult. Future LBP implementation trials in general practice should consider other novel strategies to measure patient outcomes. This could include strategies to improve patient recruitment, for example regular electronic reminders in their clinic computer systems, telephone reminders from the research team, and meaningful incentives [81], [82]. Researchers should also consider, where possible, measuring outcomes that are routinely collected and may not require individual patient consent.

Given our failure to recruit patient participants, we introduced alternative outcomes to measure the effect of the intervention. These included: i) behavioural simulation where GPs were asked to decide whether or not they would refer for x-ray, refer for other imaging, provide advice to stay active, or provide advice regarding bed rest in response to a simulated clinical situation using patient vignettes; and ii) behaviour on referrals for plain x-ray and CT scans using administrative data. These outcomes have limitations.

Behavioural simulation measures, including the use of vignettes, have previously been demonstrated to be valid measures of clinical practice and have good concordance with standardised patients and medical record abstraction [83], [84]. However, the strength of the association between behavioural simulation and clinical behaviour measures is uncertain [85]. Further, like other self-report measures, the responses may be influenced by social desirability. Although we didn’t conduct formal empirical testing of the vignettes, we carried out some assessment of content validity. As is recommended in developing measurement scales [86], we piloted the vignettes with people in the same group who were to receive the vignettes in the trial, that is, practising GPs. Further, the results from the trial itself provide some information about construct validity, since we expected that intention, as measured via vignette responses, would differ between the intervention and control groups. Given this occurred, this provides us with some confidence that the vignette responses discriminate between groups. However, it is unclear whether the observed effects for these outcomes in our trial would impact on actual GP clinical behaviour or patient outcomes.

The clinical behaviour outcomes measured via the administrative data were aggregated across all Medicare-funded patients who consulted the GP in the period 12 months post intervention delivery. It was not possible from these data to separate the population of acute LBP patients and the imaging specific to this population, that is, the data included all patients and all low back-related imaging. This raises two important issues which lead to difficulties in interpreting the effects from this administrative data.

First, we cannot be sure that the observed intervention effects (including the estimates which fall within the confidence limits) are reflective of changes in practice for the population of interest. For example, it is possible that GPs may reduce imaging for those who present with chronic LBP, or those who present with fracture, but not those with acute non-specific LBP. Second, if we assume practice change did occur only in patients presenting with acute non-specific LBP, the measurement error from the inclusion of other populations (chronic LBP, fracture, and other conditions), is likely to have attenuated the observed effects toward a value of no effect.

Despite limitations regarding interpretation of the administrative data, these data do have some advantages. A strength of administrative data recorded on all patients is that selection bias issues, that often arise in cluster randomised trials through differential recruitment of patient participants by those who are aware of their allocation status, are minimised [87]. Further, although not the focus of the intervention in this trial, routine imaging for chronic LBP is not recommended in international guidelines [88], so if a reduction of imaging in this population occurred then this would also be consistent with best practice. Finally, even though an x-ray is recommended when a GP suspects a fracture in a patient who presents with acute LBP, the prevalence of fracture in people presenting with acute LBP in Australian primary care is very low. In a cohort study conducted in Australia of 1,172 patients presenting to primary care with acute LBP, only eight fractures were detected (0.7%) [89]. Hence, we expect that the prevalence of fracture would be equally low in the patients represented in the administrative data we collected, and the influence of any x-rays that were ordered appropriately for this condition would be low. So overall, we have some confidence that a reduction of imaging in the intervention group GPs, measured by these data, indicates that the intervention would be effective.

There was some baseline imbalance in GP characteristics which may be associated with the outcome, and therefore modify the estimated intervention effects. A larger proportion of intervention group GPs indicated having undertaken LBP continuing education in the past year, while a larger proportion of control group GPs indicated having a special interest in LBP. An Australian study demonstrated that GPs who reported a special interest in LBP was associated with beliefs about management contrary to the best available evidence [90]. While all analyses adjusted for this latter pre-specified confounder, we did not adjust for LBP continuing education. Some post hoc sensitivity analyses on the administrative outcomes indicated that adjustment for LBP continuing education moved the effect estimates further from the null (results available on request). Therefore, the reported effect estimates for these outcomes, may provide a more conservative estimate of intervention effect.

Engaging healthcare clinicians to participate in research is difficult and challenging [91]. Of the general practices approached for participation, approximately 5% were recruited, and then only 61% of GPs in the intervention group were able to attend the intervention workshops. This limits the generalisability of our findings. A culture shift may be necessary in order for future implementation research to be feasible and for the results to be relevant to the broader healthcare community.

We attempted to maximise attendance at the workshops by offering an incentive to attend (continuing professional development points) and by holding the workshops on different days, times, and locations. The DVD only included the information provision (didactic) sections of the workshop and therefore the DVD did not achieve the aim of the intervention of delivering the full suite of behaviour change techniques, which required small group discussion and interaction with simulated patients. Also, considering the poor uptake of the DVD in the GPs unable to attend the workshop, our experience suggests that DVDs are not the answer for delivering this type of intervention. In future trials, other delivery modes could be considered including remote access to rurally-located GPs and online delivery, but keeping in mind the likely trade-off between achieving increased participation and the additional cost of doing so.

Practice change may require a suite of interventions with different clinicians taking up and responding to different interventions. However, this intervention was purposefully designed to be thorough and thereby required six hours of workshop attendance by the participating GPs in the intervention arm. If this thorough, theory-driven, intervention resulted in only small changes in GPs’ practice intention when compared to simple dissemination of the guideline, then it is difficult to anticipate less intense interventions being more successful.

There was some loss to follow-up for all outcome variables, which has the potential to result in some bias in the estimated intervention effects. Eleven and nine GPs were lost to follow-up in the intervention and control groups respectively (see Figure 1). Of these, four GPs in each of the intervention groups provided reasons for dropping out which would seem to be unrelated to the outcomes (i.e. moving practice, family illness, poor health, and death). This leaves seven and five GPs from the intervention and control groups respectively, for whom there is the potential that their outcomes may differ compared with those who remained in the trial. However, given these GPs are relatively few in number, the potential impact of this loss to follow-up on the intervention effects may be minor. Due to missing confounder information, there was some further missing outcome data in the fitted models. Some post-hoc sensitivity analyses of the imaging referral outcomes, comparing intervention effects estimated from models with all available outcomes versus models with the reduced subset arising from missing confounders, yielded similar estimates of intervention effect (results available on request).

Only a small fraction of GPs invited to participate in the trial agreed to do so. We do not have information on characteristics of those who did not agree to participate, and so are unable to examine whether the participating GPs differed in factors that may modify the effect of the intervention.

Finally, some care is needed when interpreting the results because of the number of outcomes analysed. It is likely that some of the statistically significant results arise through chance.

### Conclusion

An intervention based on a behaviour change theoretical framework, and evidence, compared to simple guideline dissemination alone, improved GP adherence to an evidence-based guideline measured by patient vignettes. No important change was demonstrated in most other behavioural constructs measured by questionnaire. The effect estimates for lumbar imaging were in the direction of a reduction in imaging rates in the intervention group, but the differences between groups were not statistically significant, and the potential importance of the effects was difficult to interpret. Overall, the intervention led to small changes in GP intention to practice in a manner that is consistent with an evidence-based guideline, but it did not result in statistically significant changes in actual behaviour measured via administrative data. More research is required to evaluate the effectiveness of theory-based interventions designed to improve the adherence to evidence-based guidelines for LBP management in primary care, examining actual GP behaviours in clinical practice.

## Supporting Information

Checklist S1 **CONSORT Checklist.**
(PDF)

File S1 **Tables S1 to S4.**
(PDF)

Protocol S1 **IMPLEMENT Trial Protocol.**
(PDF)

Questionnaire S1 **GP final questionnaire and scoring key.**
(PDF)
