# Supplementary material for: Evaluation of a Theory-Informed Implementation Intervention for the Management of Acute Low Back Pain in General Medical Practice: The IMPLEMENT Cluster Randomised Trial
Source: PLoS One. 2013 Jun 13;8(6):e65471. doi: 10.1371/journal.pone.0065471 (PMC3681882; doi:10.1371/journal.pone.0065471)
Supplement: File S1 — Tables S1 to S4. (PDF) [file pone.0065471.s002.pdf]

Table S1: Effect of the intervention on the behavioural constructs for the behaviours 'managing patients without referral for plain x-ray' and 'advising patients to stay active' (adjustment for design strata only)

Variable			Follow-up		Adjusted effect estimate[2]	(95% CI)	p-value
	Intervention group No. practices (no. GPs)	Control group	Intervention group Mean (SD)[1]	Control group Mean (SD)[1]			
**Managing patients without referral for plain x-ray**							
Behavioural intention (generalised)	34 (41)	38 (42)	6.2 (0.67)	5.8 (1.22)	0.2	(-0.1, 0.6)	0.205
Behavioural intention (performance)	34 (41)	37 (41)	9.4 (0.77)	8.6 (1.30)	0.8*	(0.4, 1.1)	0.000
Attitudes (direct)	34 (41)	38 (42)	6.2 (1.00)	5.8 (0.99)	0.4	(-0.0, 0.8)	0.066
Attitudes (indirect)	34 (41)	38 (42)	5.4 (0.84)	4.8 (0.97)	0.4*	(0.0, 0.7)	0.027
Subjective norms (direct)	34 (41)	38 (42)	4.7 (0.88)	4.6 (1.38)	-0.1*	(-0.5, 0.3)	0.467
Subjective norms (indirect)	34 (41)	38 (42)	4.9 (0.86)	4.5 (1.04)	0.2*	(-0.1, 0.5)	0.210
Perceived behavioural control (direct)	34 (41)	38 (42)	5.3 (1.08)	5.3 (0.94)	0.0*	(-0.3, 0.4)	0.854
Perceived behavioural control (indirect)	34 (40)	38 (42)	1.4 (7.71)	-0.2 (7.91)	1.0*	(-2.2, 4.2)	0.552
Beliefs about professional role	34 (41)	38 (42)	5.9 (0.95)	5.4 (1.14)	0.3	(-0.1, 0.7)	0.122
Knowledge (No. (%))	34 (41)	38 (42)	31 (76%)	30 (71%)	1.21[1]	(0.45, 3.26)	0.713
Beliefs about capabilities (red/yellow flags)	34 (41)	38 (42)	6.0 (0.95)	5.8 (1.23)	0.1*	(-0.3, 0.6)	0.509
Beliefs about capabilities (reassure)	34 (41)	38 (42)	6.0 (0.65)	5.6 (1.06)	0.3*	(-0.0, 0.6)	0.074
**Advising patients to stay active**							
Behavioural intention (generalised)	34 (41)	38 (42)	6.4 (0.66)	6.0 (1.24)	0.2	(-0.1, 0.5)	0.220
Behavioural intention (performance)	34 (41)	38 (42)	9.5 (1.61)	9.0 (2.08)	0.4*	(-0.2, 1.1)	0.178
Attitudes (direct)	34 (41)	38 (42)	6.3 (1.04)	6.3 (0.98)	-0.1	(-0.5, 0.4)	0.806
Attitudes (indirect)	34 (41)	38 (42)	5.2 (0.73)	4.9 (0.83)	0.2*	(-0.1, 0.5)	0.195
Subjective norms (direct)	34 (41)	38 (42)	4.9 (0.98)	4.6 (1.15)	0.2*	(-0.1, 0.6)	0.233
Subjective norms (indirect)	34 (41)	38 (42)	4.7 (0.89)	4.4 (1.03)	0.3*	(-0.1, 0.7)	0.104
Perceived behavioural control (direct)	34 (41)	38 (42)	5.8 (0.81)	5.7 (0.97)	0.1*	(-0.2, 0.4)	0.330
Perceived behavioural control (indirect)	34 (41)	38 (42)	15.9 (16.66)	13.1 (13.93)	3.4	(-2.4, 9.1)	0.251
Beliefs about professional role	34 (41)	38 (42)	6.4 (0.62)	6.3 (0.73)	0.1	(-0.2, 0.3)	0.612
Knowledge (No. (%))	34 (41)	38 (42)	39 (95%)	38 (90%)	1.67	(0.23, 11.98)	0.609
Environment context	34 (41)	38 (42)	5.7 (1.25)	5.7 (1.38)	-0.0*	(-0.5, 0.5)	0.985
Memory	34 (41)	38 (42)	5.3 (1.50)	5.0 (1.62)	0.3	(-0.2, 0.9)	0.240
Fear-avoidance beliefs	34 (41)	38 (42)	7.6 (4.23)	10.0 (4.10)	-2.5*	(-4.1, -0.9)	0.002

[1] Only general practitioners who provided both baseline and follow-up measures of the behavioural constructs are included in the calculation of the means.
[2] Adjusted difference in means estimated from marginal linear regression models using GEEs with an exchangeable correlation structure and robust variance estimation to allow for clustering within general practices. For models where the estimated within cluster correlation was negative (indicated by *), the model was refitted assuming an independent correlation structure. All models adjusted for the design strata (number of GPs per practice, location of practice [metropolitan or rural/remote]) and confounders specified prior to undertaking the analysis (age of GP (years), special interest in LBP, baseline measure of the behavioural construct).
[3] Adjusted odds ratio estimated from marginal logistic regression models using GEEs with an exchangeable correlation structure and robust variance estimation to allow for clustering within general practices. For models where the estimated within cluster correlation was negative (indicated by *), the model was refitted assuming an independent correlation structure. Models adjusted for the same variables as in footnote 1.

Table S2: Effect of the intervention on adherence to the guideline for the behaviours x-ray referral, imaging referral, advice re activity and bed rest, as measured by the vignettes (adjustment for design strata only)

Variable	Intervention group	Control group	Intervention group adherence		Control group adherence		Adjusted Odds Ratio[1]	(95% CI)	p-value
	No. practices (no. GPs)		No.	(%)	No.	(%)			
X-ray adherence[2]	34 (41)	38 (43)	136/164	(83)	119/172	(69)	2.11	(1.21, 3.68)	0.009
Imaging adherence[2]	34 (41)	38 (43)	129/164	(79)	96/172	(56)	2.96	(1.80, 4.88)	0.000
Activity adherence[2]	34 (41)	38 (43)	131/164	(80)	90/172	(52)	3.62	(1.72, 7.59)	0.001
Bed rest adherence[3]	34 (41)	38 (43)	163/164	(99)	168/171	(98)	2.91*	(0.30, 27.83)	0.354

[1] Adjusted Odds Ratios estimated from logistic models fitted using GEEs with an exchangeable correlation structure and robust variance estimation to allow for clustering within general practices. For models where the estimated within cluster correlation was negative (indicated by *), the model was refitted assuming an independent correlation structure.

[2] Model adjusted for the design strata (number of GPs per practice, location of practice [metropolitan or rural/remote]).

[3] Model not adjusted for design strata due to limited events of non-adherence.

Table S3: Effect of the intervention on imaging referral (adjustment for design strata only)

Variable	Intervention group follow-up[1]		Control group follow-up[2]		Incident rate ratios[3]	(95% CI)	p-value
	No. referrals	Rate/ 1000 patients	No. referrals	Rate/1000 patients			
X-ray referral	706	8.6	831	9.7	0.89*	(0.65, 1.22)	0.453
CT-scan referral	502	6.1	553	6.4	0.89	(0.63, 1.26)	0.509
X-ray or CT-scan referral	1208	14.7	1384	16.1	0.90*	(0.69, 1.16)	0.410

[1] No. Practices = 37 and No. GPs = 47; Total number of Medicare patients seen by GPs in intervention group = 82,392.

[2] No. Practices = 39 and No. GPs = 43; Total number of Medicare patients seen by GPs in control group = 85,899.

[3] Incident rate ratios estimated from negative binomial models fitted using GEEs with an exchangeable correlation structure and robust variance estimation to allow for clustering within general practices. For models where the estimated within cluster correlation was negative (indicated by *), the model was refitted assuming an independent correlation structure. Models adjusted for the design strata (number of GPs per practice, location of practice [metropolitan or rural/remote].

Table S4: Estimates of intra-cluster correlation coefficients (ICC)*

	ICC	Bias-corrected (95% CI)
Imaging referral		
X-ray referral	0.004	(0.003, 0.006)
CT-scan referral	0.003	(0.002, 0.005)
Vignettes		
X-ray adherence	0.048	(0.000, 0.138)
Imaging adherence	0.068	(0.000, 0.180)
Activity adherence	0.398	(0.265, 0.550)
Bed rest adherence	0.000	(0.000, 0.051)

* ICC point estimates are calculated from ANOVA. Confidence intervals for the ICCs were bootstrapped using the combination of the bootstrap and loneway commands in Stata [60]. Bootstrapping allowed for clustering of observations within general practices (using both the *cluster()* and *idcluster()* options). Bias corrected 95% confidence intervals were calculated from 1000 replicates. The ANOVA model constrains the ICC to be zero.
