# Supplementary material for: Evaluation of a Theory-Informed Implementation Intervention for the Management of Acute Low Back Pain in General Medical Practice: The IMPLEMENT Cluster Randomised Trial
Source: PLoS One. 2013 Jun 13;8(6):e65471. doi: 10.1371/journal.pone.0065471 (PMC3681882; doi:10.1371/journal.pone.0065471)
Supplement: Protocol S1 — IMPLEMENT Trial Protocol. (PDF) [file pone.0065471.s003.pdf]

Implementation Science

BioMed Central

Study protocol

Open Access

IMPLEmenting a clinical practice guideline for acute low back pain evidence-based manageMENT in general practice (IMPLEMENT): Cluster randomised controlled trial study protocol

Joanne E McKenzie*[1], Simon D French[1], Denise A O'Connor[1], Jeremy M Grimshaw[2,3], Duncan Mortimer[4,5], Susan Michie[6], Jill Francis[7], Neil Spike[8], Peter Schattner[8], Peter M Kent[8,9,10], Rachelle Buchbinder[9,10] and Sally E Green*[1]

Address: [1]Monash Institute of Health Services Research, Monash University, Melbourne, Australia, [2]Clinical Epidemiology Program, Ottawa Health Research Institute, Ottawa, Canada, [3]Department of Medicine, University of Ottawa, Ottawa, Canada, [4]Centre for Health Economics, Faculty of Business & Economics, Monash University, Melbourne, Australia, [5]Health Economics, Division of Health Sciences, University of South Australia, Adelaide, Australia, [6]Department of Psychology, University College London, UK, [7]Health Services Research Unit, University of Aberdeen, Scotland, UK, [8]School of Primary Health Care, Monash University, Australia, [9]Monash Department of Clinical Epidemiology, Cabrini Hospital, Australia and [10]Department of Epidemiology and Preventive Medicine, Monash University, Australia

Email: Joanne E McKenzie* - joanne.mckenzie@med.monash.edu.au; Simon D French - simon.french@med.monash.edu.au; Denise A O'Connor - denise.oconnor@med.monash.edu.au; Jeremy M Grimshaw - jgrimshaw@ohri.ca; Duncan Mortimer - duncan.mortimer@buseco.monash.edu.au; Susan Michie - s.michie@ucl.ac.uk; Jill Francis - j.francis@abdn.ac.uk; Neil Spike - neil.spike@med.monash.edu.au; Peter Schattner - peter.schattner@med.monash.edu.au; Peter M Kent - peter.kent@med.monash.edu.au; Rachelle Buchbinder - rachelle.buchbinder@med.monash.edu.au; Sally E Green* - sally.green@med.monash.edu.au

* Corresponding authors

Published: 22 February 2008

Implementation Science 2008, **3**:11 doi:10.1186/1748-5908-3-11

Received: 20 December 2007
Accepted: 22 February 2008

This article is available from: http://www.implementationscience.com/content/3/1/11

© 2008 McKenzie et al; licensee BioMed Central Ltd.
This is an Open Access article distributed under the terms of the Creative Commons Attribution License (http://creativecommons.org/licenses/by/2.0), which permits unrestricted use, distribution, and reproduction in any medium, provided the original work is properly cited.

Abstract

Background: Evidence generated from reliable research is not frequently implemented into clinical practice. Evidence-based clinical practice guidelines are a potential vehicle to achieve this. A recent systematic review of implementation strategies of guideline dissemination concluded that there was a lack of evidence regarding effective strategies to promote the uptake of guidelines. Recommendations from this review, and other studies, have suggested the use of interventions that are theoretically based because these may be more effective than those that are not. An evidence-based clinical practice guideline for the management of acute low back pain was recently developed in Australia. This provides an opportunity to develop and test a theory-based implementation intervention for a condition which is common, has a high burden, and for which there is an evidence-practice gap in the primary care setting.

Aim: This study aims to test the effectiveness of a theory-based intervention for implementing a clinical practice guideline for acute low back pain in general practice in Victoria, Australia. Specifically, our primary objectives are to establish if the intervention is effective in reducing the percentage of patients who are referred for a plain x-ray, and improving mean level of disability for patients three months post-consultation.

Methods/Design: This study protocol describes the details of a cluster randomised controlled trial. Ninety-two general practices (clusters), which include at least one consenting general

practitioner, will be randomised to an intervention or control arm using restricted randomisation. Patients aged 18 years or older who visit a participating practitioner for acute non-specific low back pain of less than three months duration will be eligible for inclusion. An average of twenty-five patients per general practice will be recruited, providing a total of 2,300 patient participants. General practitioners in the control arm will receive access to the guideline using the existing dissemination strategy. Practitioners in the intervention arm will be invited to participate in facilitated face-to-face workshops that have been underpinned by behavioural theory. Investigators (not involved in the delivery of the intervention), patients, outcome assessors and the study statistician will be blinded to group allocation.

Trial registration: Australian New Zealand Clinical Trials Registry ACTRN012606000098538 (date registered 14/03/2006).

Background

Research in many areas of health care has consistently demonstrated variability between the recommendations of evidence-based clinical practice guidelines (CPGs) and actual clinical practice [1]. Despite the research to date, we know little of the most effective ways to change clinical practice to implement practices we know to be effective and to remove from practice those shown to be ineffective, or even harmful. There is little evidence on which to base our plans for implementing current and future CPGs.

The most up-to-date evaluation of specific implementation strategies is a systematic review of guideline dissemination and implementation strategies that summarises the findings of 235 studies, 39 percent of which are in primary care [2]. The majority of the included studies report some improvement in care with the use of an implementation strategy. The review authors concluded that change is possible when a well-designed intervention is used; however, no single intervention is superior for all changes in all settings. They found more evidence for clinician-oriented interventions (education, reminders, feedback) than for those aimed at the organisation or the patient. They found very little information on patients' outcomes or economic assessment of the implementation strategies. Few studies used any theoretical basis to inform intervention development and the review suggested that behavioural theories should be tested to enhance our understanding of factors that influence guideline implementation strategies.

Recently, a CPG for acute low back pain (LBP) was developed and published in Australia [3]. At any one time, 26% of Australians have LBP, and 79% of Australians will experience it at some time in their lives [4]. LBP is the most frequent musculoskeletal condition managed by general practitioners (GPs) in Australia, and is the sixth most frequent reason for consulting a GP [5]. The direct and indirect cost of LBP in Australia in 2001 was estimated to total $9.175 billion (AUD) [6].

The management of people with acute LBP in general practice has been shown to have only reasonable concordance to CPG-recommended management in Australia [7]. Despite being well informed about, and agreeing with, acute LBP guidelines, many primary care practitioners do not adhere to their recommendations [8-12]. Reasons cited for non-adherence in these studies include patients' preference for non-evidence-based care (*e.g.*, x-rays) and lack of generalisability to general practice.

The aim of the CPG is to inform clinicians of the appropriate management of acute non-specific LBP. Acute non-specific LBP is defined by the guideline as pain present for less than three months, located in the lumbar and/or sacral regions of the spine with no 'red flags' present [3]. We have chosen two key messages from the CPG to target for this study that we consider are representative of the guideline as a whole. One relates to diagnosis of acute LBP and states that diagnostic x-rays are rarely necessary. Plain x-rays for acute non-specific LBP are of limited diagnostic value, expose people to unnecessary ionising radiation, and provide no benefits in physical function, pain, or disability [3]. This key message was chosen because there is evidence that plain x-rays are over utilised in the management of acute LBP in general practice. In Australia, 28% (95% confidence interval: 18% to 39%) of patients with acute LBP receive an x-ray [7] and up to 48% in the United States and Europe [8-10,13,14]. There is also wide variation of x-ray utilisation among GPs [14]. The other key message relates to providing advice to remain active. This key message was chosen because it is the only recommended therapy in the CPG with Level I evidence.

Implementing change in practice has been attempted by a number of direct and indirect methods [2]. While our project will utilise direct methods, including GP education, indirect methods such as mass media campaigns have been previously conducted and evaluated in Victoria, Australia [15-18].

We are aware of eight previous randomised controlled trials designed to change the behaviour of GPs in their management of LBP [19-26]. These trials have used various interventions including educational outreach, multi-faceted intervention including workshops and printed educational material, dissemination and, audit and feedback. The trials had varying success in changing certain behaviours of the included practitioners and provide some information about effective methods of reducing x-ray referral. However, these studies do not provide comprehensive information about successful change of other behaviours, and none utilised a behavioural theory-based intervention strategy, measured patient outcomes, or conducted a cost-effectiveness analysis.

The recently released Australian National Health and Medical Research Council (NHMRC)-endorsed evidence-based CPG for acute LBP management provides an opportunity to assess the effects of a targeted behavioural theory-based implementation strategy for use in general medical practice. This trial will assess the effectiveness of the implementation strategy both at the GP and patient levels, and also assess the cost-effectiveness of the strategy. Successful implementation of these guidelines may reduce the morbidity and societal costs of acute LBP. The trial will contribute to our knowledge about the processes underlying the effects of implementation strategies attempting to change clinical practice behaviour.

This publication is an abridged version of the full protocol. The full protocol provides more extensive justification for decisions made regarding the design and analysis of the trial. The full protocol is available from the authors on request. Details of changes made to the full protocol will be documented as amendments, also available on request. Additionally, a completed checklist of items from the CONSORT statement for cluster randomised trials [27] for the sections: title and abstract, introduction, and methods, is available as Additional File 1.

Trial objectives

The objectives of this study are to test the effectiveness of a theory-based strategy for implementing a CPG for acute LBP in general practice. We plan to measure outcomes at both the GP and patient levels to test if the intervention is effective in changing GP behaviour, and if this results in benefits for the patient. Specifically, our primary objectives are to establish if the intervention is effective in:

1. Reducing the percentage of patients who are referred for a plain x-ray for acute LBP within the three month period post initial consultation [practitioner level change].

2. Improving mean level of disability for patients three months post consultation [patient level change].

Secondary objectives include testing whether the intervention results in changes in secondary GP level outcomes, and secondary patient participant level outcomes. Secondary GP level outcomes include measurement of GP behaviours (providing advice on activity/bed rest and referral for any imaging), behavioural constructs (the GPs' attitudes, beliefs, knowledge, and intentions towards behaving in a manner consistent with the guideline's key messages) and the GPs' fear-avoidance beliefs surrounding LBP. Secondary patient level outcomes include usual pain, fear-avoidance beliefs, and whether the patient received an x-ray. We will also determine the cost-effectiveness of the developed implementation strategy that includes measuring quality of life and health service utilisation. This publication details the design and analysis of the trial. A companion paper in *Implementation Science* details the methods for the economic evaluation [28].

Methods

Trial design

This study is a cluster randomised controlled trial (CRCT), with the clusters being practices including one or more GPs. Practices will be randomly allocated to receive the intervention or control. Randomisation at the practice level has been chosen because this will minimise contamination that may occur if individual patients were randomised, and the GPs were required to manage patients in the control and experimental groups concurrently [29]. Additionally, since all GPs within the same practice will receive the same intervention, this will minimise potential contamination that may occur if GPs within the same practice were randomly allocated to different intervention groups.

Eligibility and recruitment

Recruitment of general practices

A sampling frame of 1,000 general practices within the state of Victoria, Australia, will be obtained from the publishing company Australasian Medical Publishing Company (AMPCo). From this, a random sample of 400 general practices will be selected and approached for participation in the CRCT. GPs at these practices will receive a postcard with a short description of the study, followed by an invitation letter. In addition, GPs will also be contacted by telephone by one of the research staff. A sample of 400 practices has been chosen, since we expect that the response rate may be less than 50% [30]. Practices that meet the eligibility criteria will be randomised. If we are unable to recruit the required sample of 92 practices from the 400 approached, a further group of 400 practices will be randomly sampled from the sampling frame. We will then assess the need to obtain more data from AMPCo based on the response rate to this initial process. To increase the awareness of the study, notices will be placed in the newsletters of the Divisions of General Practice

(Victoria) and other printed/electronic newsletters. If more than 92 practices agree to participate, we will include the first 92 that respond. When one GP in a practice agrees to participate and the practice is included, we will then send follow-up letters to the other GPs in the same practice informing them that the practice is included, encouraging them to participate, and allowing them to object to the practice participating if they wish.

Strategies to promote participation of general practices into the trial include offering professional development points, providing access to experts, and providing GPs with skills in the management of acute LBP.

Recruitment of patient participants

After the intervention has been delivered, included practices will be provided with posters and pamphlets that explain the study and provide contact details of the research team. It will be up to the individual patient to initiate their enrolment in the trial. Posters will be placed in the participating practices that will explain the study in plain language and suggest to potential patient participants to contact the research team for more information. The patient will then indicate to the receptionist their interest in the study and the receptionist will give them a copy of the explanatory statement and the consent form. These will outline the details of the study and will explain how to contact the research team. Additionally, general practice reception staff will be briefed about the trial to facilitate patient recruitment.

This recruitment strategy has been chosen to reduce the potential risk of selection bias that may occur if GPs, practice nurses, or receptionists were to approach patients. However, it is unclear whether this passive approach will be successful. The recruitment rate will be reviewed one month into patient recruitment by the investigators. If the recruitment rate is not adequate at that time, a more proactive approach will be instigated. This may include measures such as frequent visits to general practices from the IMPLEMENT staff and handing out of pamphlets to all patients attending the practice by the practice receptionist.

Applying the eligibility criteria

Screening of potential general practices will be conducted by one member of the research team over the telephone via discussion with the practice manager.

Screening of potential patient participants will be a two-step process to prevent possible selection bias. A research assistant who is blinded to the general practice group allocation will initially speak with potential patient participants. Demographic information, including their GP's name, will be collected. The patient participant will then be transferred to a clinician (SF or SG) who will determine their eligibility for inclusion in the trial. Before speaking with the clinician, the research assistant will explain to the patient participant why they should not inform the clinician of their GP's name.

Inclusion criteria

General practices will be included if the following criteria are met:

1) The practice principal agrees to the practice being involved in the CRCT.

2) At least one GP within the practice provides written informed consent.

3) Practice support staff are willing to facilitate patient recruitment.

GPs will be included if they provide written informed consent.

Patients attending enrolled practices will be included if the following criteria are met:

1) They attend a consenting GP for acute non-specific LBP (duration of less than three months).

2) Provide written informed consent.

3) Are 18 years of age or older.

4) Are able to read and understand spoken English to a level where they can read the study information sheet, complete the consent form, and respond to the telephone delivered questionnaire. This will be assessed by the outcome assessor at the time of screening.

Exclusion criteria

General practices will be excluded if a GP objects to their practice being included in the trial.

Individual GPs will be excluded if they work at more than one of the general practices included in the trial.

Patients attending enrolled practices will not be eligible for inclusion if any of the following criteria are met. This will be determined by one of the clinical investigators (SG or SF) over the telephone. Note that these criteria reflect the clinical scope of the acute LBP CPG [3].

1) Radicular leg pain is present. We define this as leg pain that is described as shooting, lancinating, or electric in quality, extends below the knee, has a dermatomal distribution and may be associated with paraesthesia.

2) They have had previous spinal surgery.

3) 'Red flags' are present alerting the possibility of serious conditions such as malignancy, infection, or fracture.

4) Pregnancy.

Randomisation and allocation concealment

General practices meeting the inclusion criteria will be randomly allocated to receive either the intervention or control. Restricted randomisation will be used to reduce the probability of baseline imbalance. Within stratum, one-half of the practices will be randomised to the intervention group, and the other half to the control using computer-generated random numbers. Four strata will be defined by the number of GPs per practice (two levels) and whether the practice is in a rural or metropolitan area. A statistician independent of the study will implement the randomisation. They will be provided with only general practice codes and stratification variables. Allocation will be concealed from the investigators until baseline data has been collected from GPs.

Blinding

Investigators (not involved in the delivery of the intervention), patients, outcome assessors, and study statistician (JM) will be blinded to group allocation until the statistical analysis has been completed. Due to the nature of the intervention, it is not possible to blind the GPs to group allocation. However, the GPs will only be told that they are to receive one of two interventions, one being information only and the other attending facilitated small group educational workshops. The success of blinding will not be evaluated.

Interventions

Control group

The control group will receive access to the guideline as per the CPG's existing dissemination strategy. A printed copy of the guideline and a written reminder of how to access the electronic version of the CPG will be sent to control group practices.

Intervention group

The GPs randomised to the intervention arm will receive an intervention specifically designed to address the perceived barriers and enablers for implementation of the CPG. In phase one of this project, focus group interviews were conducted with GPs in Victoria, Australia, underpinned by a theoretical framework grounded in behavioural theory [31]. Thematic analysis was used to map the identified barriers and enablers to the theoretical domains that are used for understanding and facilitating behaviour change. Multiple barriers to implementing the key messages of the guideline were identified in discussion with GPs. The principal barriers included beliefs about negative consequences of practising in a manner consistent with the guideline recommendations; beliefs about patient preferences or expectations inconsistent with the guideline recommendations; beliefs about limitations in their capabilities and skills to practice in a manner consistent with the guideline; limitations in their knowledge and the knowledge of their patients to practice in a manner consistent with the guideline; and barriers in the social and environmental context in which they operate that made behaviour consistent with the guideline more difficult. Results from analyses of the focus group interviews will be published in a separate publication.

The intervention will consist of a combination of behaviour change techniques. These techniques will be utilised throughout the workshops including providing instruction; modelling/demonstrating the behaviour by a peer expert; providing information on consequences; prompt barrier identification; time management; prompt specific goal setting; rehearsal; persuasive communication; and scripting [32]. These specific techniques have been chosen because they are considered the best approach to address the barriers and enablers to the CPG's implementation [33]. The intervention will concentrate on delivering the CPG's key messages, namely that diagnostic x-rays are rarely necessary in the management of acute LBP and that advising patients to remain active reduces pain and disability. The intervention will consist of facilitated face-to-face small group workshops. There will be a pre-course reflective activity where the GPs will document their management of a series of patients that present to them with acute LBP over the two weeks preceding the workshop. There will be two workshops of three hours each, or one workshop of six hours depending on the preference of the GP. These workshops will be directed by a trained GP facilitator and will involve a combination of didactic lectures and small group discussions. A detailed description of the targeted implementation strategy, including the development process, will be reported in a separate publication. We also plan to evaluate the fidelity of delivery of the intervention to assess the extent to which the intervention, as delivered, was faithful to the intervention as planned [34]. A mixed-methods approach will be employed based on a recent project that evaluated fidelity of delivery of a physical activity intervention [35]. We plan to report this evaluation in a separate publication.

For GPs in the intervention group who cannot attend the workshops, a DVD will be provided to them. The DVD will include film footage of the workshops and electronic resources related to LBP management.

Timing of recruitment, intervention delivery and follow-up

The intervention workshops and delivery of DVDs span a three month period (26 June 2007 – 26 September 2007), during which materials are sent to the GPs in the control group (23 August 2007). Patient participant recruitment begins at least six weeks post intervention or control group delivery (28 October 2007) and continues for five months.

Study outcomes

Primary outcome measures

The primary GP level outcome is whether the GP referred the patient for a plain x-ray within a three month period post-patient enrolment. The primary patient level outcome is LBP-specific disability three months from enrolment (Table 1).

Referral for x-ray has been chosen as the primary practitioner level outcome for two major reasons. First, the intervention will focus on the delivery of two key messages from the CPG, with one of the messages being that diagnostic x-rays are rarely necessary in the management of acute LBP. Referral for x-ray measures the effectiveness of the intervention at the practitioner level. Second, this outcome can be reliably determined, since evidence of referral for x-ray is expected to be recorded in the patient's medical record.

The second clinical practice that will form the focus of the intervention is providing advice to patients to stay active. Evidence that underpins this recommendation suggests that staying active results in a beneficial effect on pain, rate of recovery, and function. Disability has been chosen as the primary patient level outcome because this has the potential to have a substantial impact on the patient's quality of life, and we believe this is of importance in informing health care decisions. In addition, evidence suggests that short-term disability (measured at three months) is a risk factor for long-term disability [36], thus improving short-term disability may provide long-term benefits. This would include obvious benefits to the patients, and potential benefits for health service utilisation and to employers.

Table 1: Outcome measures

Outcome	Data collection method	Follow-up schedule	Source	Level at which data are collected
**GP level**				
X-ray referral (process)[1,2]	Data abstraction	3 months	Patient medical record	Patient
Advice to stay active (process)	Telephone interview	7 days after consultation	Patient	Patient
Advised bed rest (process)	Telephone interview	7 days after consultation	Patient	Patient
Any imaging referral (process)[3]	Data abstraction	3 months	Patient medical record	Patient
FAB-Q[4]	Questionnaire	Baseline, 12 months	GP	GP
Measurement of behavioural constructs[5]	Questionnaire	Baseline, 12 months	GP	GP
**Patient level**				
Roland-Morris Disability Questionnaire (RDQ)[1,6]	Telephone interview	7 days and 3 months after consultation	Patient	Patient
Usual pain[7]	Telephone interview	7 days and 3 months after consultation	Patient	Patient
X-ray occurred	Telephone interview	3 months	Patient	Patient
FAB-Q[4]	Telephone interview	3 months	Patient	Patient
Assessment of Quality of Life (AQoL)	Telephone interview	7 days and 3 months after consultation	Patient	Patient
Health Service Utilisation Items	Telephone interview	7 days and 3 months after consultation	Patient	Patient

[1] Primary outcome.
[2] Includes either evidence of referral for x-ray or evidence that an x-ray has been taken (*e.g.*, copy of x-ray film with GP name).
[3] Includes either evidence of referral for any imaging or evidence that imaging has occurred.
[4] FAB-Q physical activity subscale [61]. The original scale will be used in patient participants. A modified version will be used for GP participants with details of the modifications available in the full protocol. Details of the reliability, validity and responsiveness are available in Waddell *et al.* [61] and George *et al.* [62].
[5] Table 2 provides details on the behavioural constructs.
[6] The RDQ measures 24 activity limitations due to back pain. Reliability and validity for use over the phone reported in Roland *et al.* [63].
[7] Measured using an eleven point numerical rating scale (0 – 10) with the question "On a scale of zero to 10, zero being no pain and 10 being pain as bad as it can be, where would you rate your usual pain today?". Reliability and validity for its use over the phone is reported in Von Korff *et al.* [64].

Disability will be measured with the Roland-Morris Disability Questionnaire (RDQ) [37] which is one of the standard questionnaires used in LBP research. This was chosen above the Oswestry Disability Questionnaire, another standard LBP-specific disability questionnaire, for two reasons. First, it is the preferred questionnaire to use when the mode of interviewing is carried out by telephone, because of ease of use. Second, it is recommended for use in populations where individuals have comparatively lower disability levels, as is expected for patient participants included in this trial [38].

It is common in trials assessing the effectiveness of interventions aimed at increasing the uptake of evidence in clinical practice to only include outcome measures of change in practitioner behaviour [39]. It was considered important to measure patient level outcomes in this trial because it was unclear whether the implementation strategy would result in a change in the patient's health status. Trials which underpin the key message from the guideline on providing advice to stay active differ in regard to the interventions employed, delivery of the intervention and control arms, and have only shown small beneficial effects for the outcomes pain, rate of recovery and function [3].

Secondary outcome measures

Secondary GP level outcomes include whether or not the GP provided advice on activity or bed rest or referred for any type of imaging, scores on measures of behavioural constructs (Table 2), and GPs' fear-avoidance beliefs (Table 1). Secondary patient participant level outcomes include usual pain, fear-avoidance beliefs, and whether the patient received a plain x-ray. Additionally, health-related quality of life and health service utilisation will be measured and this data used in the economic evaluation [28]. Rationale for the selection of secondary outcomes is provided in the full protocol, available on request.

Data quality assurance

Details of data quality assurance for the trial are available in the full protocol. In brief, methods used to enhance the quality of the data will include: daily checking for accuracy and completion of data forms; range checks; and cross-form consistency. Double data entry will be used for the GP questionnaires (Table 1). All telephone interviews at the patient participant level will be recorded and a fraction of these will be checked using a continuous sampling plan [40,41]. In addition, a continuous sampling plan will be used to check a sample of data abstracted from patient medical records.

Table 2: Behavioural constructs

Domains	Explanation	Domain measured for behaviour	
		X-ray[1]	Activity[2]
Behavioural intention[3]	Whether the GP intends to engage in the behaviour.		
Generalised		✓	✓
Performance		✓	✓
Attitude[4]	Whether the GP is in favour of performing the behaviour.		
Direct		✓	✓
Indirect		✓	✓
Subjective norm[4]	How much the GP feels social pressure to engage in the behaviour.		
Direct		✓	✓
Indirect		✓	✓
Perceived behavioural control[4]	Whether the GP feels in control of the behaviour.		
Direct		✓	✓
Indirect		✓	✓
Beliefs about capabilities	Whether the GP is confident in performing the behaviour.	✓	✓
Beliefs about professional role	Whether the GP feels it is their professional responsibility to perform the behaviour.	✓	✗
Knowledge	Whether the GP has knowledge of the behaviour.	✓	✓
Memory	Whether the GP remembers to perform the behaviour.	✗	✓
Environmental context	Whether the GP feels the environmental context supports performance of the behaviour.	✗	✓

[1] Managing patients without referral for plain x-ray.
[2] Providing advice to stay active.
[3] Domain measured using generalised method (*e.g.*, by asking GPs about their strength of intention to perform the behaviour) and performance method (*e.g.*, asking GPs about how often they intend to perform the behaviour).
[4] Domain measured directly (*e.g.*, by asking GPs about their overall attitude) and indirectly (*e.g.*, by asking about specific behavioural beliefs).
Details of the number of items measuring each domain, for each behaviour, and measures of reliability and validity of the constructs are available in the full protocol.

Sample size

The primary process and patient outcomes are x-ray referral and LBP-specific disability as measured by the RDQ [37]. In the calculation of sample size for these outcomes, adjustment needs to be made for the clustered nature of the design. The inflation factor used to achieve this is determined from the average cluster size and the intra-cluster correlation (ICC) [42]. Empirical research has suggested ICCs of the order 0.10 for process measures and 0.05 for patient outcomes in primary care [43]. These have been used in the following calculations. It is estimated that 28% of current GP consultations for acute LBP in Australia result in a lumbar spine x-ray [7]. Interventions comparing standard CPG dissemination with no intervention control groups have shown improvements in care of approximately 8% [2]. We therefore anticipate a decrease in the percentage of x-ray prescription in the control group of this magnitude. We consider a reduction of 10% to be clinically important. Therefore, if the percentage of x-ray prescription at the end of the study in the control group is 20%, a sample size of 37 general practices per arm, with an average of 25 patients per practice, will be sufficient to detect an absolute decrease in the percentage of x-ray prescription of 10% (to 10%) or more with 90% power. This assumes a significance level of 5% and an ICC = 0.10.

This sample size will be sufficient to detect a clinically important difference of at least two points between groups in the RDQ with at least 99% power, assuming a standard deviation of six [44,45], significance level of 5%, and an ICC = 0.05.

Allowing for 20% attrition in practices (equivalent to 450 patient participants), we plan to initially recruit 46 practices per arm, with an average of 25 patients per practice, providing a total of 2,300 patients. These sample size calculations are likely to be conservative because, due to a lack of prior information, stratification has not been incorporated into the calculations.

Analyses

Analysis subsets

The primary analysis of the data in this CRCT will be analysed using the principle of intention-to-treat (ITT). This is appropriate for a CRCT such as ours, since an analysis which allows for non-compliance by GPs and patients is more likely to provide an estimate of intervention effect that is more reflective of actual clinical practice [46,47].

Requirements for an ideal ITT analysis are: full compliance with the randomised intervention; no missing responses; and follow-up on all participants [46]. In a CRCT the potential for non-adherence to the intervention and loss to follow-up are more complex than an individual RCT, because it can occur at multiple levels. For example, in our CRCT, GPs within general practices may not comply with the intervention, GPs may withdraw, and even general practices may discontinue participation. At the patient level, there may also be non-adherence to advice provided by the GP, patient withdrawal or loss to follow-up.

Non-adherence to the intervention is likely to reduce its potential effectiveness, and provide a conservative estimate of intervention effect compared to what would be expected if there was full compliance [46]. Loss to follow-up is likely to lead to biased estimates of intervention effect, particularly when there is differential drop out between intervention arms that is related to the intervention itself. We plan to implement procedures to minimise loss to follow-up and withdrawal at the patient, GP, and general practice level. Additionally, through modelling, we will investigate the effect of missing data (details available in the full protocol). Where possible, we will collect information on reasons for patient, GP, and general practice withdrawal.

For the primary outcomes (Table 1), a secondary per-protocol analysis will be carried out to estimate the effect of the intervention for the subgroup of GPs who complied with the intervention. Compliance to the intervention is defined as attendance of at least one workshop.

Primary analysis

Comparisons of outcomes between the intervention and control groups will be made by appropriately adjusting for the correlation that occurs within general practices. Several common approaches for analysing data from CRCTs include: adjustment of standard statistical tests; analysis at the cluster level; and advanced statistical modelling techniques that can use both data recorded at the individual and cluster level. Modelling techniques are attractive since potential confounding variables at the patient, GP, and general practice level can be adjusted for. This is especially useful in CRCTs since the chance of baseline imbalance in prognostic factors is likely to be higher than in trials where individuals are randomised, because the number of clusters is generally fewer than the number of individuals.

Two common types of modelling are marginal and cluster-specific. In a cluster-specific approach the dependence between observations within a cluster is explicitly modelled, while in a marginal model this dependence is treated as a nuisance parameter. No consensus exists on which method is preferred, and both methods have advantages and disadvantages [48]. However, we plan to use marginal modelling using generalised estimating equations (GEEs). This modelling method has been chosen above a cluster-specific approach primarily for the fol-

lowing reasons. First, the cluster-specific random effects models that have been developed for non-normally distributed data present considerable computational difficulties which can result in biased estimates [29]. Second, the interpretation of the estimated regression coefficients from a GEE have a population interpretation, the same as that of regression coefficients from general linear models [49,50]. They measure the expected change in a response for those in the intervention group, as compared to those in the control group [51]. For cluster-specific models, the interpretation is conditional on the cluster. Neuhaus [52] suggested that interpretation of the intervention effect estimated from cluster-specific models in a CRCT is difficult because all participants within a cluster receive the same intervention. Because of this, Neuhuas remarked that marginal models are conceptually preferable for estimating cluster-level effects such as intervention status.

A disadvantage of using GEEs is that only one level of clustering can be modelled. This CRCT includes three levels of data: general practices; GP participants; and patient participants. It is possible to allow for clustering at the general practice level or the GP level. We have chosen to cluster at the general practice level because this is the unit of randomisation, and adjusting for clustering at this level will appropriately account for correlation that may occur between GPs within the same practice.

For both continuous and binary outcomes, we plan to fit GEEs with an exchangeable correlation structure, where responses from the same cluster are assumed to be equally correlated [53]. Additionally, we intend to use robust variance estimation that will provide valid standard errors even if the within-cluster correlation structure has been incorrectly specified [29,54]. For binary outcomes, a logit link function will be used.

Our primary analysis of outcomes will include adjustment for stratification variables (number of GPs per practice and rurality) and pre-specified potential confounders (available in the full version of the protocol). All pre-specified confounders will be included in the models even when no baseline imbalance exists. This approach has been chosen because confounder selection strategies that are based on collected data, (*e.g.*, selecting confounders using preliminary statistical tests) result in models with poor statistical properties such as incorrect type I error rates [55-57]. In addition, for continuous outcomes that are collected at both baseline and follow-up (measurement of behavioural constructs and FAB-Q at the GP level), we will include the baseline measure as a covariate in the model. This method will appropriately adjust for any baseline imbalance that we may observe, and provides the most powerful analysis [57,58].

The potential confounders have been selected through discussion with LBP and implementation researchers, and from published research. For statistical parsimony, the number of included confounders for each outcome has been limited to those which we surmise are the most important. However, we will investigate the impact of additional confounders through modelling as part of the exploratory analyses (details available in the full version of the protocol). Additionally, estimates of intervention effect estimated from models including only the stratification variables will be presented.

Estimates of intervention effect from these models will yield odds ratios. For interpretability, we plan to also provide estimates of risk ratios using a simple formula that will utilise the odds ratios estimated from these models [59]. For each outcome, the estimate of intervention effect and its 95% confidence interval will be provided. For the primary outcomes we plan to provide estimates of the coefficient of ICC and their 95% confidence intervals.

No adjustment will be made for multiple testing. All tests will be two-sided and carried out at the 5% level of significance.

Any future change to the statistical methods outlined in this analysis strategy will be documented with full justification as an amendment to the full protocol.

Publication policy

The chief investigator will be responsible for ensuring timely production of a scientific manuscript at the completion of the trial. The results from the trial will be submitted for publication irrespective of outcome. All authors and the trial management committee, consisting of the authors listed on this publication, will review and approve the final manuscript prior to submission. The final trial results will be submitted for publication in a major international medical journal for wide dissemination. Reporting of this trial will adhere to the CONSORT statement and its extension to CRCTs [27,60]. Additional publications documenting development of the intervention, intervention fidelity and investigating mediating effects between primary and secondary outcomes are planned.

Ethical review

Ethical approval for this trial was obtained from the Monash University Standing Committee on Ethics in Research involving Humans (2006/047). The investigators will ensure that the trial will be conducted in compliance with this protocol, the Guideline for Good Clinical Practice (CPMH/ICH/135/95) and the Australian National Statement on Ethical Conduct of Research Involving Humans.

Competing interests

Sally Green, Jeremy Grimshaw and Susan Michie are all members of the editorial board of Implementation Science. The remaining authors declare that they have no competing interests.

Authors' contributions

JM, SF, DO, JG, DM and SG conceptualised and designed the study and secured funding. PK and RB provided input on the design. JM, SF, DO, JG, SM, JF, NS, PS and SG designed the intervention. JM, SF and DM wrote the full protocol, with comments provided by the other authors. JM and SF wrote the first draft of this manuscript. All authors contributed to revisions of this manuscript, have read and approved the final manuscript, and take public responsibility for its content.

Additional material

Additional file 1

Completed CONSORT checklist for the IMPLEMENT CRCT study protocol. The completed checklist of items from the CONSORT statement for cluster randomised trials for the sections: title and abstract, introduction, and methods.
Click here for file
[http://www.biomedcentral.com/content/supplementary/1748-5908-3-11-S1.pdf]

Acknowledgements

We are grateful to the following reviewers who all provided valuable statistical and content review of our full protocol: Marion Campbell (Health Services Research Unit, University of Aberdeen, United Kingdom), Obioha Ukoumunne (Murdoch Childrens Research Institute and the University of Melbourne Department of Paediatrics, Australia), Monica Taljaard (Ottawa Health Research Institute, Clinical Epidemiology Program, Ottawa, Canada), Glenn Pransky (Center for Disability Research, Liberty Mutual Research Institute for Safety, Hopkinton, Massachusetts, USA) and Gertrude Bekkering (Department of Social Medicine, University of Bristol, United Kingdom). We thank the IMPLEMENT Advisory Committee, consisting of representatives of divisions of general practice in Victoria (Australia) and researchers, for their input into various design and planning issues for the study: David Clarke (Centre for Psychological and Behavioural Medicine, Monash University, Australia), Lynne Cooper and John Siemienowicz (Mornington Peninsula Division of General Practice), Claire Harris (Centre for Clinical Effectiveness, Southern Health, Australia), Neil Hearnden (Royal Australian College of General Practitioners), Jill Kelly (Whitehorse Division of General Practice), Chris Maher (School of Physiotherapy, University of Sydney, Australia), Mary Mathews (Monash Division of General Practice), Bill Newton (General Practice Divisions Victoria), Anne Peek (Dandenong Division of General Practice), Carolyn Searle (North West Melbourne Division of General Practice), Renzo Sgarbossa (Western Melbourne Division of General Practice), Melanie Virtue (Knox Division of General Practice). We acknowledge the contribution to the conception of the study provided by the late Professor Jeffrey Richards.

The trial is funded by the NHMRC by way of a Primary Health Care Project Grant (334060). JF has 50% of her time funded by the Chief Scientist Office of the Scottish Government Health Directorate and 50% by the University of Aberdeen. PK is supported by a NHMRC Health Professional Fellowship (384366) and RB by a NHMRC Practitioner Fellowship (334010). JG holds a Canada Research Chair in Health Knowledge Transfer and Uptake. All other authors are funded by their own institutions. The NHMRC has had no involvement in the study design, preparation of the manuscript, or the decision to submit the manuscript.

References

1. Grol R: **Successes and failures in the implementation of evidence-based guidelines for clinical practice.** *Med Care* 2001, **39:**46-54.
2. Grimshaw JM, Thomas RE, MacLennan G, Fraser C, Ramsay CR, Vale L, Whitty P, Eccles MP, Matowe L, Shirran L, Wensing M, Dijkstra R, Donaldson C: **Effectiveness and efficiency of guideline dissemination and implementation strategies.** *Health Technol Assess* 2004, **8:**iii-iv. 1–72.
3. Australian Acute Musculoskeletal Pain Guidelines Group (AAMPGG): *Evidence-based management of acute musculoskeletal pain* 2003 [http://nhmrc.gov.au]. Brisbane: Australian Academic Press
4. Walker BF, Muller R, Grant WD: **Low back pain in Australian adults: prevalence and associated disability.** *J Manipulative Physiol Ther* 2004, **27:**238-244.
5. Britt H, Miller G, Charles J, Pan Y, Valenti L, Henderson J, Bayram C, O'Halloran J, Knox S: **General practice activity in Australia 2005–06.** In *General practice series no 19 AIHW cat no GEP 19* Canberra: Australian Institute of Health and Welfare; 2007.
6. Walker BF, Muller R, Grant WD: **Low back pain in Australian adults: the economic burden.** *Asia Pac J Public Health* 2003, **15:**79-87.
7. McGuirk B, King W, Govind J, Lowry J, Bogduk N: **Safety, efficacy, and cost effectiveness of evidence-based guidelines for the management of acute low back pain in primary care.** *Spine* 2001, **30:**2615-2622.
8. Di Iorio D, Henley E, Doughty A: **A survey of primary care physician practice patterns and adherence to acute low back problem guidelines.** *Arch Fam Med* 2000, **9:**1015-1021.
9. Gonzalez-Urzelai V, Palacio-Elua L, Lopez-de-Munain J: **Routine primary care management of acute low back pain: adherence to clinical guidelines.** *Eur Spine J* 2003, **12:**589-594.
10. Frankel BS, Moffett JK, Keen S, Jackson D: **Guidelines for low back pain: changes in GP management.** *Fam Pract* 1999, **16:**216-222.
11. Schers H, Braspenning J, Drijver R, Wensing M, Grol R: **Low back pain in general practice: reported management and reasons for not adhering to the guidelines in The Netherlands.** *Br J Gen Pract* 2000, **50:**640-644.
12. Little P, Smith L, Cantrell T, Chapman J, Langridge J, Pickering R: **General practitioners' management of acute back pain: A survey of reported practice compared with clinical guidelines.** *BMJ* 1996, **312:**485-488.
13. Carey TS, Garrett J: **Patterns of ordering diagnostic tests for patients with acute low back pain. The North Carolina Back Pain Project.** *Ann Intern Med* 1996, **125:**807-814.
14. Freeborn DK, Shye D, Mullooly JP, Eraker S, Romeo J: **Primary care physicians' use of lumbar spine imaging tests: effects of guidelines and practice pattern feedback.** *J Gen Intern Med* 1997, **12:**619-625.
15. Buchbinder R, Jolley D, Wyatt M: **Population based intervention to change back pain beliefs and disability: three part evaluation.** *BMJ* 2001, **322:**1516-1520.
16. Buchbinder R, Jolley D: **Population based intervention to change back pain beliefs: three year follow up population survey.** *BMJ* 2004, **328:**321.
17. Buchbinder R, Jolley D: **Effects of a media campaign on back beliefs is sustained 3 years after its cessation.** *Spine* 2005, **32(11):**1323-1330.
18. Buchbinder R, Jolley D: **Improvements in general practitioner beliefs and stated management of back pain persist 4.5 years after the cessation of a public health media campaign.** *Spine* 2007, **32:**E156-162.
19. Robling MR, Houston HL, Kinnersley P, Hourihan MD, Cohen DR, Hale J, Hood K: **General practitioners' use of magnetic resonance imaging: an open randomized trial comparing telephone and written requests and an open randomized**

controlled trial of different methods of local guideline dissemination. *Clin Radiol* 2002, **57:**402-407.
20. Dey P, Simpson CW, Collins SI, Hodgson G, Dowrick CF, Simison AJ, Rose MJ: **Implementation of RCGP guidelines for acute low back pain: a cluster randomised controlled trial.** *Br J Gen Pract* 2004, **54:**33-37.
21. Engers AJ, Wensing M, van Tulder MW, Timmermans A, Oostendorp RA, Koes BW, Grol R: **Implementation of the Dutch low back pain guideline for general practitioners: a cluster randomized controlled trial.** *Spine* 2005, **30:**559-600.
22. Oakeshott P, Kerry SM, Williams JE: **Randomized controlled trial of the effect of the Royal College of Radiologists' guidelines on general practitioners' referrals for radiographic examination.** *Br J Gen Pract* 1994, **44:**197-200.
23. Schectman JM, Schroth WS, Verme D, Voss JD: **Randomized controlled trial of education and feedback for implementation of guidelines for acute low back pain.** *J Gen Intern Med* 2003, **18:**773-780.
24. Eccles M, Steen N, Grimshaw J, Thomas L, McNamee P, Soutter J, Wilsdon J, Matowe L, Needham G, Gilbert F, Bond S: **Effect of audit and feedback, and reminder messages on primary-care radiology referrals: a randomised trial.** *Lancet* 2001, **357:**1406-1409.
25. Bishop PB, Wing PC: **Knowledge transfer in family physicians managing patients with acute low back pain: a prospective randomized control trial.** *Spine J* 2006, **6:**282-288.
26. Kerry S, Oakeshott P, Dundas D, Williams J: **Influence of postal distribution of the Royal College of Radiologists' guidelines, together with feedback on radiological referral rates, on X-ray referrals from general practice: a randomized controlled trial.** *Fam Pract* 2000, **17:**46-52.
27. Campbell MK, Elbourne DR, Altman DG, CONSORT group : **CONSORT statement: extension to cluster randomised trials.** *BMJ* 2004, **328:**702-708.
28. Mortimer D, French SD, McKenzie JE, O'Connor DA, Green SE: **Protocol for economic evaluation alongside the IMPLEMENT cluster randomised controlled trial.** *Implementation Science* 2008, **3(1):**12.
29. Ukoumunne OC, Gulliford MC, Chinn S, Sterne JA, Burney PG: **Methods for evaluating area-wide and organisation-based interventions in health and health care: a systematic review.** *Health Technol Assess* 1999, **3:**iii-92.
30. Yallop JJ, McAvoy BR, Croucher JL, Tonkin A, Piterman L: **Primary health care research – essential but disadvantaged.** *Med J Aust* 2006, **185:**118-120.
31. Michie S, Johnston M, Abraham C, Lawton R, Parker D, Walker A, "Psychological Theory" Group: **Making psychological theory useful for implementing evidence based practice: a consensus approach.** *Qual Saf Health Care* 2005, **14:**26-33.
32. Abraham C, Michie S: **A taxonomy of behavior change techniques used in interventions.** *Health Psychol* in press.
33. Francis J, Michie S, Johnston M, Hardeman W, Eccles M: **How do behaviour change techniques map on to psychological constructs? Results of a consensus process.** *Presented at the European Health Psychology annual conference, Galway, Ireland* 2005.
34. Bellg AJ, Borrelli B, Resnick B, Hecht J, Minicucci DS, Ory M, Ogedegbe G, Orwig D, Ernst D, Czajkowski S, Treatment Fidelity Workgroup of the NIH Behavior Change Consortium: **Enhancing treatment fidelity in health behavior change studies: best practices and recommendations from the NIH Behavior Change Consortium.** *Health Psychol* 2004, **23:**443-451.
35. Hardeman W, Michie S, Fanshawe T, Prevost A, McLoughlin K, Kinmonth A: **Fidelity of delivery of a physical activity intervention: predictors and consequences.** *Psychology and Health* 2008, **12:**11-24.
36. Kent PM, Keating JL: **Can we predict poor recovery from recent-onset nonspecific low back pain? A systematic review.** *Man Ther* 2008, **13:**12-28.
37. Roland M, Morris R: **A study of the natural history of back pain. Part I: development of a reliable and sensitive measure of disability in low-back pain.** *Spine* 1983, **8:**141-144.
38. Bombardier C: **Outcome assessments in the evaluation of treatment of spinal disorders: summary and general recommendations.** *Spine* 2000, **25:**3100-3103.
39. Hakkennes S, Green S: **Measures for assessing practice change in medical practitioners.** *Implement Sci* 2006, **1:**29.
40. King DW, Hazelwood M: **Quantifying Gains in Data Quality for Sampling Plans Used in Clinical Trial Monitoring.** *Drug Inf J* 2003, **37:**135-141.
41. King DW, Lashley R: **A quantifiable alternative to double data entry.** *Control Clin Trials* 2000, **21:**94-102.
42. Donner A, Birkett N, Buck C: **Randomization by cluster. Sample size requirements and analysis.** *Am J Epidemiol* 1981, **114:**906-914.
43. Campbell M, Grimshaw J, Steen N: **Sample size calculations for cluster randomised trials. Changing Professional Practice in Europe Group (EU BIOMED II Concerted Action).** *J Health Serv Res Policy* 2000, **5:**12-16.
44. Bombardier C, Hayden J, Beaton DE: **Minimal clinically important difference. Low back pain: outcome measures.** *J Rheumatol* 2001, **28:**431-438.
45. Hay EM, Mullis R, Lewis M, Vohora K, Main CJ, Watson P, Dziedzic KS, Sim J, Minns Lowe C, Croft PR: **Comparison of physical treatments versus a brief pain-management programme for back pain in primary care: a randomised clinical trial in physiotherapy practice.** *Lancet* 2005, **365:**2024-2030.
46. Heritier SR, Gebski VJ, Keech AC: **Inclusion of patients in clinical trial analysis: the intention-to-treat principle.** *Med J Aust* 2003, **179:**438-440.
47. Hollis S, Campbell F: **What is meant by intention to treat analysis? Survey of published randomised controlled trials.** *BMJ* 1999, **319:**670-674.
48. Mollison J: **Use of cluster randomised trials in implementation research.** In *Doctor of Philosophy* University of Aberdeen; 2002.
49. Diggle PJ, Heagerty P, Liang KY, Zeger SL: *Analysis of Longitudinal Data* Second edition. New York: Oxford University Press; 2002.
50. Hu FB, Goldberg J, Hedeker D, Flay BR, Pentz MA: **Comparison of population-averaged and subject-specific approaches for analyzing repeated binary outcomes.** *Am J Epidemiol* 1998, **147:**694-703.
51. Klar N, Donner A: **Current and future challenges in the design and analysis of cluster randomization trials.** *Stat Med* 2001, **20:**3729-3740.
52. Neuhaus JM: **Statistical methods for longitudinal and clustered designs with binary responses.** *Stat Methods Med Res* 1992, **1:**249-273.
53. Horton NJ, Lipsitz SR: **Review of software to fit generalised estimating equation regression models.** *Am Stat* 1999, **53:**160-169.
54. Fitzmaurice GM, Laird NM, Ware JH: **Marginal models: Generalized Estimating Equations (GEE).** In *Applied longitudinal analysis* Edited by: Balding DJ, Cressie NAC, Fisher NI, Johnstone IM, Kadane JB, Molenberghs G, Ryan LM, Scott DW, Smith AFM, Teugels JL. Hoboken, New Jersey: John Wiley & Sons; 2004.
55. Permutt T: **Testing for imbalance of covariates in controlled experiments.** *Stat Med* 1990, **9:**1455-1462.
56. Pocock SJ, Assmann SE, Enos LE, Kasten LE: **Subgroup analysis, covariate adjustment and baseline comparisons in clinical trial reporting: current practice and problems.** *Stat Med* 2002, **21:**2917-2930.
57. Senn SJ: **Covariate imbalance and random allocation in clinical trials.** *Stat Med* 1989, **8:**467-475.
58. Altman DG: **Covariate imbalance, adjustment for.** In *Encyclopedia of biostatistics* Edited by: Armitage P, Colton T. New York: J. Wiley; 1998:1000-1005.
59. Zhang J, Yu KF: **What's the relative risk? A method of correcting the odds ratio in cohort studies of common outcomes.** *JAMA* 1998, **280:**1690-1691.
60. Altman DG, Schulz KF, Moher D, Egger M, Davidoff F, Elbourne D, Gotzsche PC, Lang T, CONSORT GROUP (Consolidated Standards of Reporting Trials): **The revised CONSORT statement for reporting randomized trials: explanation and elaboration.** *Ann Intern Med* 2001, **134(8):**663-694.
61. Waddell G, Newton M, Henderson I, Somerville D, Main CJ: **A Fear-Avoidance Beliefs Questionnaire (FABQ) and the role of fear-avoidance beliefs in chronic low back pain and disability.** *Pain* 1993, **52:**157-168.
62. George SZ, Fritz JM, McNeil DW: **Fear-avoidance beliefs as measured by the fear-avoidance beliefs questionnaire: change in fear-avoidance beliefs questionnaire is predictive of change in self-report of disability and pain intensity for patients with acute low back pain.** *Clin J Pain* 2006, **22:**197-203.

63. Roland M, Fairbank J: **The Roland-Morris Disability Questionnaire and the Oswestry Disability Questionnaire.** *Spine* 2000, **25:**3115-3124.
64. Von Korff M, Jensen MP, Karoly P: **Assessing global pain severity by self-report in clinical and health services research.** *Spine* 2000, **25:**3140-3151.

Publish with **BioMed Central** and every scientist can read your work free of charge

"BioMed Central will be the most significant development for disseminating the results of biomedical research in our lifetime."

Sir Paul Nurse, Cancer Research UK

Your research papers will be:

- available free of charge to the entire biomedical community
- peer reviewed and published immediately upon acceptance
- cited in PubMed and archived on PubMed Central
- yours — you keep the copyright

Submit your manuscript here:
http://www.biomedcentral.com/info/publishing_adv.asp

BioMedcentral
