# Supplementary material for: Evaluation of a Theory-Informed Implementation Intervention for the Management of Acute Low Back Pain in General Medical Practice: The IMPLEMENT Cluster Randomised Trial
Source: PLoS One. 2013 Jun 13;8(6):e65471. doi: 10.1371/journal.pone.0065471 (PMC3681882; doi:10.1371/journal.pone.0065471)
Supplement: Questionnaire S1 — GP final questionnaire and scoring key. (PDF) [file pone.0065471.s004.pdf]

The management of acute low-back pain
in general practice

Office use only : | GP ID : | | CLINIC ID: | |

PART A

1 First name ______________________ Surname ______________________

2 Email: ____

3 Would you like us to send you a copy of the results of this study when it is published? Yes No

The management of acute low-back pain
in general practice

Office use only : | GP ID : | | CLINIC ID: | |

PART B

The following questions **all** relate to **patients with acute, non-specific low-back pain**. This means patients with uncomplicated low-back pain of less than three months duration and without any serious underlying pathology suspected.

Please circle a number (from 1 to 7) for each item below that best represents your views about plain x-rays.

**1**	People who are important to me professionally think that I should manage patients with acute non-specific low-back pain without referring for plain x-ray	***Strongly disagree***	1	2	3	4	5	6	7	***Strongly agree***
**2**	For me, managing these patients without referring for plain x-ray is easy	***Strongly disagree***	1	2	3	4	5	6	7	***Strongly agree***
**3**	I expect to manage these patients without referring for plain x-ray	***Strongly disagree***	1	2	3	4	5	6	7	***Strongly agree***
**4**	Other GPs would approve of me managing these patients without referring for plain x-ray	***Strongly disagree***	1	2	3	4	5	6	7	***Strongly agree***
**5**	I have complete control over the decision on whether I manage these patients without referring for plain x-ray	***Strongly disagree***	1	2	3	4	5	6	7	***Strongly agree***
**6**	I think WorkCover would approve of me managing compensable patients with acute non-specific low-back pain without referring for plain x-ray	***Strongly disagree***	1	2	3	4	5	6	7	***Strongly agree***
**7**	Managing these patients without referring for plain x-ray is an appropriate part of my work as a GP	***Strongly disagree***	1	2	3	4	5	6	7	***Strongly agree***
**8**	I intend to manage these patients without referring for plain x-ray	***Strongly disagree***	1	2	3	4	5	6	7	***Strongly agree***
**9**	If I manage these patients without referring for plain x-ray I might miss important underlying pathology	***Unlikely***	1	2	3	4	5	6	7	***Likely***
**10**	Whether or not I manage these patients without referring for plain x-ray is entirely up to me	***Strongly disagree***	1	2	3	4	5	6	7	***Strongly agree***
**11**	I plan to manage these patients without referring for plain x-ray	***Strongly disagree***	1	2	3	4	5	6	7	***Strongly agree***
**12**	I am confident that I can manage these patients without referring for plain x-ray	***Strongly disagree***	1	2	3	4	5	6	7	***Strongly agree***
**13**	Radiologists would approve of me managing these patients without referring for plain x-ray	***Strongly disagree***	1	2	3	4	5	6	7	***Strongly agree***
**14**	If I manage these patients without referring for plain x-ray I might increase my vulnerability to legal action	***Unlikely***	1	2	3	4	5	6	7	***Likely***
**15**	It is part of my professional role as a GP to refer these patients for plain x-ray in order to reassure them that there is nothing seriously wrong	***Strongly disagree***	1	2	3	4	5	6	7	***Strongly agree***
**16**	Patients who come to me with acute non-specific low-back pain expect that I will manage them without referring for plain x-ray	***Strongly disagree***	1	2	3	4	5	6	7	***Strongly agree***

The management of acute low-back pain in general practice

**17**	Thinking about your next 10 patients presenting with acute non-specific low-back pain, how many of them will you refer for plain x-ray?	☐ …of 10 [*please specify*]								
**18**	I am confident that I can reassure patients with acute non-specific low-back pain that there is nothing seriously wrong without referring for plain x-ray	***Strongly disagree***	1	2	3	4	5	6	7	***Strongly agree***
**19**	If I manage these patients without referring for plain x-ray I feel that I am protecting them from unnecessary radiation	***Unlikely***	1	2	3	4	5	6	7	***Likely***
**20**	If I don't refer these patients for plain x-ray they will go to another GP/health care provider who will	***Unlikely***	1	2	3	4	5	6	7	***Likely***
**21**	It is in accordance with Medicare policy that I manage these patients without referring for plain x-ray	***Strongly disagree***	1	2	3	4	5	6	7	***Strongly agree***
**22**	It is expected of me that I manage these patients without referring for plain x-ray	***Strongly disagree***	1	2	3	4	5	6	7	***Strongly agree***
**23**	If I refer these patients for plain x-ray I will have to spend extra time explaining results to them that are not relevant to their back pain	***Unlikely***	1	2	3	4	5	6	7	***Likely***
**24**	Patients with acute non-specific low-back pain come to the consultation with an expectation that they should have a plain x-ray	***Unlikely***	1	2	3	4	5	6	7	***Likely***
**25**	When patients come to the consultation with an expectation that they should have a plain x-ray, I am… [*circle a response to indicate how likely you are*] …to manage them without referring for plain x-ray	***Less likely***	1	2	3	4	5	6	7	***More likely***
**26**	I feel under pressure to manage these patients without referring for plain x-ray	***Strongly disagree***	1	2	3	4	5	6	7	***Strongly agree***
**27**	When I am uncertain of my diagnosis a plain x-ray is useful	***Unlikely***	1	2	3	4	5	6	7	***Likely***
**28**	Being uncertain of my diagnosis makes it… [*circle a response to indicate how difficult it is*] ...to manage these patients without referring for plain x-ray	***Less difficult***	1	2	3	4	5	6	7	***More difficult***
**29**	If I manage these patients without referring for plain x-ray they will not feel reassured that there is nothing seriously wrong	***Unlikely***	1	2	3	4	5	6	7	***Likely***

30 For me, managing patients with acute non-specific low-back pain without referring for plain x-ray is… [*circle a response for each item below*]

**a)**	***Bad practice***	1	2	3	4	5	6	7	***Good practice***
**b)**	***Harmful to the patient***	1	2	3	4	5	6	7	***Beneficial to the patient***
**c)**	***The wrong thing to do***	1	2	3	4	5	6	7	***The right thing to do***
**d)**	***Useless***	1	2	3	4	5	6	7	***Useful***
**e)**	***Not my preferred approach***	1	2	3	4	5	6	7	***My preferred approach***

The management of acute low-back pain in general practice

**31**	It is my professional role as a GP to exclude pathology in these patients using plain x-ray	***Strongly disagree***	1	2	3	4	5	6	7	***Strongly agree***
**32**	I am confident that I can identify red and yellow flags in patients presenting with acute low-back pain	***Strongly disagree***	1	2	3	4	5	6	7	***Strongly agree***

33 I am aware of the recommendation regarding referral for plain x-ray from the evidence-based guideline for managing patients with acute non-specific low-back pain. [*Please tick one box that best describes your opinion*]

YES

NO

34 Practising in a manner consistent with the evidence-based guideline for acute non-specific low-back pain means …….. [*Please tick one option below that best describes your opinion*]

a) Referring these patients for plain x-ray

b) Referring these patients for plain x-ray only when alerting features (red flags) of serious conditions are present

c) Managing these patients without referring for plain x-ray

d) Other (please specify) ____________________

____

PART C

Please circle a number (from 1 to 7) for each item below that best represents your views about advice to stay active.

1 Thinking about your next 10 patients presenting with acute non-specific low-back pain, how many of them will you advise to stay active? [ ] …of 10 [*please specify*]

**2**	For me, advising these patients to stay active is easy	***Strongly disagree***	1	2	3	4	5	6	7	***Strongly agree***
**3**	I expect to advise these patients to stay active	***Strongly disagree***	1	2	3	4	5	6	7	***Strongly agree***
**4**	The decision to advise these patients to stay active is beyond my control	***Strongly disagree***	1	2	3	4	5	6	7	***Strongly agree***
**5**	If I advise these patients to stay active, they will think that I am unsympathetic to their pain	***Unlikely***	1	2	3	4	5	6	7	***Likely***
**6**	Advising these patients to stay active is an appropriate part of my work as a GP	***Strongly disagree***	1	2	3	4	5	6	7	***Strongly agree***
**7**	I feel under pressure to advise these patients to stay active	***Strongly disagree***	1	2	3	4	5	6	7	***Strongly agree***
**8**	Patients with acute non-specific low-back pain complain of severe pain	***Unlikely***	1	2	3	4	5	6	7	***Likely***
**9**	When patients with acute non-specific low-back pain complain of severe pain, I am…*[circle a response to indicate how likely you are]*...to advise them to stay active	***Less likely***	1	2	3	4	5	6	7	***More likely***
**10**	I am confident that I can advise these patients to stay active if I want to	***Strongly disagree***	1	2	3	4	5	6	7	***Strongly agree***

The management of acute low-back pain in general practice

**11**	Sometimes I forget to advise these patients to stay active	***Strongly disagree***	1	2	3	4	5	6	7	***Strongly agree***
**12**	Advice about activity needs to be tailored to the individual	***Strongly disagree***	1	2	3	4	5	6	7	***Strongly agree***
**13**	When advice about activity needs to be tailored to the individual patient, I am… *[circle a response to indicate how likely you are]* ...to advise them to stay active	***Less likely***	1	2	3	4	5	6	7	***More likely***
**14**	I intend to advise these patients to stay active	***Strongly disagree***	1	2	3	4	5	6	7	***Strongly agree***
**15**	It is expected of me that I advise these patients to stay active	***Strongly disagree***	1	2	3	4	5	6	7	***Strongly agree***
**16**	Advising these patients to stay active is not part of my professional role	***Strongly disagree***	1	2	3	4	5	6	7	***Strongly agree***
**17**	People who are important to me professionally think that I should advise these patients to stay active	***Strongly disagree***	1	2	3	4	5	6	7	***Strongly agree***
**18**	Other GPs would approve of my giving advice to stay active to these patients	***Strongly disagree***	1	2	3	4	5	6	7	***Strongly agree***
**19**	I plan to advise these patients to stay active	***Strongly disagree***	1	2	3	4	5	6	7	***Strongly agree***
**20**	Patients with acute non-specific low-back pain will get better regardless of whether I advise them to stay active or not	***Unlikely***	1	2	3	4	5	6	7	***Likely***
**21**	Patients who come to me with acute non-specific low-back pain expect that I will advise them to stay active	***Strongly disagree***	1	2	3	4	5	6	7	***Strongly agree***
**22**	If I advise these patients to stay active, they will exacerbate their low-back pain	***Unlikely***	1	2	3	4	5	6	7	***Likely***
**23**	If I don't advise these patients to stay active, they will develop chronic pain	***Unlikely***	1	2	3	4	5	6	7	***Likely***
**24**	If I advise these patients to stay active, they will improve at a faster rate	***Unlikely***	1	2	3	4	5	6	7	***Likely***
**25**	Whether I advise these patients to stay active or not is entirely up to me	***Strongly disagree***	1	2	3	4	5	6	7	***Strongly agree***
**26**	It's difficult to find time in the consultation to advise these patients to stay active	***Strongly disagree***	1	2	3	4	5	6	7	***Strongly agree***
**27**	Patients with acute non-specific low-back pain don't ask for advice about activity in the consultation	***Unlikely***	1	2	3	4	5	6	7	***Likely***
**28**	When these patients don't ask for advice about activity in the consultation, I am… *[circle a response to indicate how likely you are]* ...to advise them to stay active	***Less likely***	1	2	3	4	5	6	7	***More likely***
**29**	If I advise these patients to stay active, they will ignore my advice anyway	***Unlikely***	1	2	3	4	5	6	7	***Likely***

30 For me, advising patients with acute non-specific low-back pain to stay active is…
[*circle a response for each item below*]

**a)**	***Bad practice***	1	2	3	4	5	6	7	***Good practice***
**b)**	***Harmful to the patient***	1	2	3	4	5	6	7	***Beneficial to the patient***
**c)**	***The wrong thing to do***	1	2	3	4	5	6	7	***The right thing to do***
**d)**	***Useless***	1	2	3	4	5	6	7	***Useful***
**e)**	***A waste of my time***	1	2	3	4	5	6	7	***A good use of my time***

31 I am aware of the recommendation regarding advice to stay active (activation) from the evidence-based guideline for managing patients with acute non-specific low-back pain. (*Please tick one box that best describes your opinion*)

YES

NO

32 Advising these patients to stay active means …
(*Please tick the one box below that best describes your opinion*)

a) Advising the patient not to lie in bed

b) Advising the patient to continue with their normal daily activities within the limits of pain

c) Advising the patient to do specific back exercises

d) Advising the patient to do general exercises (eg. walking)

e) Other (please specify) ____________________

PART D

Here are some of the things which people have told us about their acute non-specific low-back pain. For each statement please circle any number from 1 to 7 to say how much physical activities (such as bending, lifting, walking or driving) affect, or would affect, back pain:

**1**	Physical activity makes acute non-specific low-back pain worse	***Strongly disagree***	1	2	3	4	5	6	7	***Strongly agree***
**2**	Physical activity might harm people with acute non-specific low-back pain	***Strongly disagree***	1	2	3	4	5	6	7	***Strongly agree***
**3**	People with acute non-specific low-back pain should not do physical activities which (might) make their pain worse	***Strongly disagree***	1	2	3	4	5	6	7	***Strongly agree***
**4**	People with acute non-specific low-back pain cannot do physical activities which (might) make their pain worse	***Strongly disagree***	1	2	3	4	5	6	7	***Strongly agree***

PART E

This section contains four patient scenarios about patients who present to you with acute low-back pain. In the scenarios we have varied a range of features that might influence your management decisions (in regards to investigations you might order and interventions you might recommend). At the end of each scenario we ask you to indicate whether or not you would order an investigation for the patient described in the scenario, and what interventions you would recommend for this patient. The scenarios differ slightly in various elements. We are aware that the scenario format means that skills you may normally draw on, such as evaluating non-verbal clues from the patient and performing a physical examination, cannot be a factor in your assessment. Nevertheless, given this understanding, we hope that you address each scenario and answer the questions as best as you can with the information provided. We have left space for you to comment on your decisions, if you wish.

Scenario 1

A 48 year old office worker attends your clinic. He is usually very active, playing lots of sport and does regular exercise (eg jogging, gym). He has low-back pain, rated 5 out of 10. The pain started two weeks ago and is located in the low back region, right sided, no radiation. The pain is relieved by stretching his low back and using a heated wheat bag. The pain is worse after playing sport, to the point where in the last week he had to stop mid-game during basketball. He has no previous history of low-back pain. The patient thinks that an x-ray is required to “find out what is wrong”, and he is fearful that movement and activity might make the pain worse.

1. Would you order any investigations for this patient at this visit?
Please tick all that apply:

☐ None

☐ Lumbosacral plain x-ray

☐ Lumbar CT scan

☐ Lumbar MRI

☐ Other (please specify) ____________________________

2. Which interventions would you recommend to this patient at this visit?
Please tick all that apply:

☐ Bed rest for ____ days

☐ Paracetamol

☐ Non-steroidal anti-inflammatory drugs

☐ Advise the patient to do specific back exercises

☐ Advise the patient to do general exercises (eg. walking)

☐ Advise the patient to continue with their normal daily activities

☐ Manual therapy (spinal manipulative therapy, mobilisation or massage)

☐ Referral to another health care provider (eg physiotherapist, chiropractor, pain clinic, acupuncture).

If yes please specify ____________________________

☐ Other (please specify) ____________________________

If you wish to comment on your management decisions, please do so here:

____

Scenario 2

A 57 year old office worker sees you for low-back pain. She says her pain began 8 weeks ago. There was no specific incident that caused the pain. The pain is located in the lower back region, with no radiation. The pain is a dull ache (3 out of 10), with occasional sharp "twinges" with certain movements. The pain is relieved by heat and a massage from her spouse. She has no history of low-back pain. The patient is overweight (BMI 28), has mild hypertension, and a family history of type 2 diabetes. The patient rarely does any exercise. During the consultation she indicates to you that she is anxious that she may have a serious disease. The patient says "a friend had low-back pain like this and they had an x-ray and it showed that they really had something major wrong with them". She repeatedly requests an x-ray during the consultation.

1. Would you order any investigations for this patient at this visit?
Please tick all that apply:

☐ None

☐ Lumbosacral plain x-ray

☐ Lumbar CT scan

☐ Lumbar MRI

☐ Other (please specify) ____________________________

2. Which interventions would you recommend to this patient at this visit?
Please tick all that apply:

☐ Bed rest for ____ days

☐ Paracetamol

☐ Non-steroidal anti-inflammatory drugs

☐ Advise the patient to do specific back exercises

☐ Advise the patient to do general exercises (eg. walking)

☐ Advise the patient to continue with their normal daily activities

☐ Manual therapy (spinal manipulative therapy, mobilisation or massage)

☐ Referral to another health care provider (eg physiotherapist, chiropractor, pain clinic, acupuncture).

If yes please specify ____________________________

☐ Other (please specify) ____________________________

If you wish to comment on your management decisions, please do so here:

____

____

Scenario 3

A 36 year old real estate agent consults you for his low-back pain. He comes in on a very busy day at the practice and there are many patients already in the waiting room wanting to see you. The pain has been present for six weeks, starting two days after moving heavy furniture at home. Pain is described as an ache (4 to 5 out of 10). There is no radiation. He has had previous, similar episodes of low back pain that have lasted one to two weeks only. The patient has no other health concerns. The patient has seen you, or a colleague of yours in the practice, weekly over the last four weeks for their low-back pain. He is dissatisfied that he has not already been referred for an x-ray, and insists that you refer him for an x-ray now.

1. Would you order any investigations for this patient at this visit?
Please tick all that apply:

☐ None

☐ Lumbosacral plain x-ray

☐ Lumbar CT scan

☐ Lumbar MRI

☐ Other (please specify) ____________________________

2. Which interventions would you recommend to this patient at this visit?
Please tick all that apply:

☐ Bed rest for ____ days

☐ Paracetamol

☐ Non-steroidal anti-inflammatory drugs

☐ Advise the patient to do specific back exercises

☐ Advise the patient to do general exercises (eg. walking)

☐ Advise the patient to continue with their normal daily activities

☐ Manual therapy (spinal manipulative therapy, mobilisation or massage)

☐ Referral to another health care provider (eg physiotherapist, chiropractor, pain clinic, acupuncture).

If yes please specify ____________________________

☐ Other (please specify) ____________________________

If you wish to comment on your management decisions, please do so here:

____

____

Scenario 4

A 28 year old woman has suffered from low-back pain for one week. She has been unable to do her job managing a hospital cafeteria for this time. She walks slowly into your consultation room, holding her back and grimacing. She sits with a loud groan. She says she has severe low-back pain, describing it as 9 out of 10. While anxious to return to work, she feels immobilised by the pain. There is no history of trauma. The pain is in the low-back area, without radiation. On physical examination there is marked limitation of anterior flexion and tenderness in the left paraspinal region. The neurological examination is normal with straight leg raising to 90 degrees. She has had numerous episodes of back pain in the past but thinks this is the worst episode she has ever had and is very worried that whatever is causing his problem is getting worse.

1. Would you order any investigations for this patient at this visit?
Please tick all that apply:

☐ None

☐ Lumbosacral plain x-ray

☐ Lumbar CT scan

☐ Lumbar MRI

☐ Other (please specify) ____________________________

2. Which interventions would you recommend to this patient at this visit?
Please tick all that apply:

☐ Bed rest for ____ days

☐ Paracetamol

☐ Non-steroidal anti-inflammatory drugs

☐ Advise the patient to do specific back exercises

☐ Advise the patient to do general exercises (eg. walking)

☐ Advise the patient to continue with their normal daily activities

☐ Manual therapy (spinal manipulative therapy, mobilisation or massage)

☐ Referral to another health care provider (eg physiotherapist, chiropractor, pain clinic, acupuncture).

If yes please specify ____________________________

☐ Other (please specify) ____________________________

If you wish to comment on your management decisions, please do so here:

____

____

Thank you very much for your participation!

Please provide an estimate of the time taken in minutes to complete this form

The management of acute low-back pain in general practice

Scoring key for behavioural constructs (Part B, Part C and Part D)

TPB constructs	Target behaviour	Items and scale	Scoring and interpretation
Attitudes	manage these patients (patients with acute non-specific low back pain) without referring for plain x-ray	PART B<br>5 direct items (#30 a-e); 7-pt scale<br><br>6 indirect items - behavioural beliefs only (#9, 14, 19, 20, 23, 29); 7-pt scale	**Att direct** = mean of 5 items. Higher scores reflect positive attitude to target behaviour<br><br>Recode items 9, 14, 20, 29 (reverse-score).<br>**Att indirect** = mean of 6 items. NB. No outcome evaluation items included. Higher scores reflect more favourable attitudes toward target behaviour
	advise these patients (patients with acute non-specific low back pain) to stay active	PART C<br>5 direct items (#30 a-e); 7-pt scale<br><br>6 indirect items – behavioural beliefs only (#5, 20, 22, 23, 24, 29); 7-pt scale	**Att direct** = mean of 5 items. Higher scores reflect positive attitude to target behaviour<br><br>Recode items 5, 20, 22, 29 (reverse-score).<br>**Att indirect** = mean of 6 items. NB. No outcome evaluation items included. Higher scores reflect more favourable attitudes toward target behaviour
Subjective norms	manage these patients without referring for plain x-ray	PART B<br>3 direct items (#1, 22, 26); 7-pt scale<br><br>5 indirect items – normative beliefs only (#4, 6, 13, 16, 21); 7-pt scale	**SN direct** = mean of 3 items. Higher scores reflect greater social pressure to do target behaviour<br><br>**SN indirect** = mean of 5 items. NB. No motivation to comply items included
	advise these patients to stay active	PART C<br>3 direct items (#7, 15, 17) 7-pt scale<br><br>2 indirect items – normative beliefs only (#18, 21); 7-pt scale	**SN direct** = mean of 3 items. Higher scores reflect greater social pressure to do target behaviour<br><br>**SN indirect** = mean of 2 items. NB. No motivation to comply items included
Perceived behavioural control	manage these patients without referring for plain x-ray	PART B<br>4 direct items (#2, 12, 5, 10); 7-pt scale<br>- 2 items are self-efficacy (#2, 12)<br>- 2 items are controllability (#5, 10)	**PBC direct** = mean of 4 items. Higher scores reflect a greater level of control over target behaviour

		4 indirect items (#24, 27, 25, 28); 7-pt scale<br>- 2 items are control beliefs (#24, 27)<br>- 2 items are control power (#25, 28)	Recode item #25: 1=-3, 2=-2, 3=-1, 4=0, 5=+1, 6=+2, 7=+3.<br>Recode item #28: 1=+3, 2=+2, 3=+1, 4=0, 5=-1, 6=-2, 7=-3.<br><br>**PBC indirect** = (#24 x #25) + (#27 x #28) (range -42 to +42)<br>Positive scores reflect higher control over target behaviour. Negative scores indicate lower control
	advise these patients to stay active	PART C<br>4 direct items (#2, 10, 4, 25); 7-pt scale<br>- 2 items are self-efficacy (#2, 10)<br>- 2 items are controllability (#4, 25)<br><br>6 indirect items (#8, 12, 27, 9, 13, 28); 7-pt scale<br>- 3 items are control beliefs (#8, 12, 27)<br>- 3 items are control power (#9, 13, 28)	Recode item 4 (reverse-score).<br>**PBC direct** = mean of 4 items. Higher scores reflect a greater level of control over target behaviour<br><br>Recode items #9, 13, 28: 1=-3, 2=-2, 3=-1, 4=0, 5=+1, 6=+2, 7=+3.<br><br>**PBC indirect** = (#8 x #9) + (#12 x #13) + (#27 x #28) (range -63 to +63)<br>Positive scores reflect higher control over target behaviour. Negative scores indicate lower control
Behavioural intentions	manage these patients without referring for plain x-ray	PART B<br>4 direct items (#3, 8, 11, 17)<br>- 3 items are generalised intention (#3, 8, 11); 7-pt scale<br>- 1 item is intention performance (#17); 11-pt scale	**BI direct** = mean of 3 generalised intention items. Higher scores reflect a greater generalised intention to perform the target behaviour<br><br>**BIP direct** = single score from intention performance item (range 0 to 10). Higher score reflects higher behavioural intention to <u>NOT</u> perform the target behaviour (i.e. refer for plain x-ray - this is because of the manner in which the target behaviour is expressed in this item)
	advise these patients to stay active	PART C<br>4 direct items (#3, 14, 19, 1)<br>- 3 items are generalised intention (#3, 14, 19); 7-pt scale	**BI direct** = mean of 3 generalised intention items. Higher scores reflect a greater generalised intention to perform the target

		- 1 item is intention performance (#1); 11-pt scale	behaviour<br><br>**BIP direct** = single score from intention performance item (range 0 to 10). Higher score reflects higher behavioural intention to perform the target behaviour (i.e. advise patients to stay active)

**Scoring of additional items (for purpose of trial)**	**Relevant key message from guideline (i.e. x-ray or advice)**	**Items and scale**	**Scoring**
Beliefs about professional role	x-ray	PART B<br>3 items (#7, 15, 31); 7-pt scale	Recode items 15, 31 (reverse-score).<br>**Professional role summary score** = mean of 3 items.
	advice	PART C<br>2 items (#6, 16); 7-pt scale	Recode item 16 (reverse-score).<br>**Professional role summary score** = mean of 2 items.
Knowledge about key messages of the guideline	x-ray	PART B<br>2 items (#33, 34)	Individual items. The following responses indicate adequate knowledge:<br>- ‘yes’ response to item 33<br>- option b and c is selected for item 34 (scored as 1=adequate, 0=not adequate)
	advice	PART C<br>1 item (#31)<br><br>N.B. Item 32 indicates their interpretation of the key message (what advice to stay active means to them)	Individual item. A ‘yes’ response to item 31 indicates adequate knowledge
Beliefs about capabilities (to identify red and yellow flags)	x-ray	PART B<br>1 item (#32)	Individual item.
Beliefs about capabilities (to reassure patient that nothing seriously wrong with their back without referring for plain x-ray)	x-ray	PART B<br>1 item (#18)	Individual item.
Environmental context (time)	advice	PART C<br>1 item (#26)	Individual item. Recode item 26 (reverse-score).
Memory	advice	PART C<br>1 item (#11)	Individual item. Recode item 11 (reverse-score).

Fear avoidance beliefs	advice about activity	PART D<br>4 items (#1-4 in Part C)	Recode items #1 to 4: 1=0, 2=1, 3=2, 4=3, 5=4, 6=5, 7=6.<br>**Fear avoidance beliefs questionnaire physical score (FABQ-Phys) summary score** = sum of items #1-4 (min to max score is 0 to 24).<br>Interpretation: low scores = low fear avoidance beliefs.
